# Hydrogen Sulfide Biochemistry and Interplay with Other Gaseous Mediators in Mammalian Physiology

**DOI:** 10.1155/2018/6290931

**Published:** 2018-06-27

**Authors:** Alessandro Giuffrè, João B. Vicente

**Affiliations:** ^1^CNR Institute of Molecular Biology and Pathology, Rome, Italy; ^2^Instituto de Tecnologia Química e Biológica António Xavier, NOVA University of Lisbon, Av. da República (EAN), 2780-157 Oeiras, Portugal

## Abstract

Hydrogen sulfide (H_2_S) has emerged as a relevant signaling molecule in physiology, taking its seat as a bona fide gasotransmitter akin to nitric oxide (NO) and carbon monoxide (CO). After being merely regarded as a toxic poisonous molecule, it is now recognized that mammalian cells are equipped with sophisticated enzymatic systems for H_2_S production and breakdown. The signaling role of H_2_S is mainly related to its ability to modify different protein targets, particularly by promoting persulfidation of protein cysteine residues and by interacting with metal centers, mostly hemes. H_2_S has been shown to regulate a myriad of cellular processes with multiple physiological consequences. As such, dysfunctional H_2_S metabolism is increasingly implicated in different pathologies, from cardiovascular and neurodegenerative diseases to cancer. As a highly diffusible reactive species, the intra- and extracellular levels of H_2_S have to be kept under tight control and, accordingly, regulation of H_2_S metabolism occurs at different levels. Interestingly, even though H_2_S, NO, and CO have similar modes of action and parallel regulatory targets or precisely because of that, there is increasing evidence of a crosstalk between the three gasotransmitters. Herein are reviewed the biochemistry, metabolism, and signaling function of hydrogen sulfide, as well as its interplay with the other gasotransmitters, NO and CO.

## 1. Introduction

### 1.1. Brief Historical Account of Hydrogen Sulfide

Hydrogen sulfide (H_2_S) is known in popular culture as a toxic poisonous gas and a source of foul smell, such as that of rotten eggs, sewers, swamps, and volcanic sources. Ancient alchemic practices produced several preparations now known to release hydrogen sulfide, for example, the H_2_S- and ammonia-rich gases released upon distillation to obtain Liquor Hepatis (Balsam of the Soul), made from limestone and camel dung. In his treaty on workers' diseases “De Morbis Artificum,” the 17th–18th century Italian physician Bernardino Ramazzini first described painful eye inflammation often leading to secondary bacterial infection and eventually blindness in workers who cleaned privies and cesspits [1]. Ramazzini hypothesized that, when the workers stirred the excrements, an unknown volatile compound was released which, besides having the undesired health effects, was also responsible for turning black their copper and silver coins on the surface. The same toxic volatile substance, originated in the Paris sewers, was likely responsible for a series of incidents in the late 18th century, ranging from mild eye and mucous membrane inflammation to severe asphyxia. Almost at the same time, in 1777, the Swedish chemist Carl Wilhelm Scheele described the stinking substance (sulfur air) resulting from the reaction of pyrite (ferrous disulfide) with a mineral acid [2].

While long considered a poisonous substance, not only hazardous for workers but also burdensome for some industrial processes (e.g., “oil souring” in oil extraction), hydrogen sulfide was in the late 20th century discovered to be endogenously formed in humans, and in the early 21st century, it was recognized as an extremely relevant signaling molecule in physiology and general biology.

### 1.2. General Chemistry of Hydrogen Sulfide

Hydrogen sulfide is a colorless flammable gas. It is a weak acid, existing in aqueous solution in equilibrium with hydrosulfide (HS^−^) and sulfide (S^2−^), according to p*K*_a1_~7.0 (H_2_S/HS^−^) and p*K*_a2_~19 (HS^−^/S^2−^), at 25°C ([3] and references therein). At physiological pH values, the S^2−^ concentration in solution is therefore negligible. According to this equilibrium, at the physiological pH 7.4, c.a. 70% of hydrogen sulfide is in its HS^−^ form, the remainder occurring as H_2_S. In the alkaline mitochondrial matrix (pH 8.0), HS^−^ reaches 92%, while the remaining 8% corresponds to H_2_S. Contrarily, under the acidic conditions in lysosomes (pH 4.7), >99% of hydrogen sulfide is in its H_2_S form and is slightly polar, allowing it to freely diffuse across and accumulate in aqueous or hydrophobic milieu such as biological membranes. Herein, unless otherwise stated, the terms “hydrogen sulfide” or “sulfide” and the abbreviation “H_2_S” henceforth collectively designate the pool of the H_2_S, HS^−^, and S^2−^ species.

H_2_S is the sulfur species corresponding to the lowest sulfur oxidation state (−2). Sulfur, with its electronic configuration [Ne] 3s^2^ 3p^4^, is a highly redox versatile element, with oxidation states ranging from −2 to +6 (as in sulfate), due to the six valence electrons. This versatility certainly accounts for its biological usefulness and is probably related to the major role of sulfur in the emergence and evolution of life on Earth (reviewed in [4]). H_2_S is a reducing species, displaying a reduction potential of −280 mV (pH 7.0, against the standard hydrogen electrode) for the two-electron HS^−^/S^0^ redox couple (−230 mV for H_2_S/S^0^) [5], close to the values for the glutathione disulfide/glutathione (GSSG/GSH) and the cystine/cysteine redox couples.

Presently, despite major advances, the physiological H_2_S concentrations are still a matter of debate, and the posited levels have been decreasing with the increasing sophistication and accuracy of the detection methods. Currently, free H_2_S is reported to be submicromolar, although it may exist in equilibrium with a pool of labile sulfur-containing molecules that can liberate H_2_S “on demand,” that is, under particular physiological conditions [6]. To add more complexity into the role of H_2_S in human (patho)physiology, it has become clear that a great part of the signaling effects attributed to H_2_S (see below) actually results from the occurrence of persulfides and polysulfides, among other sulfur-containing molecules, that have been collectively termed “reactive sulfur species” (RSS). More details on the formation of persulfides and polysulfides, and their physiological relevance intertwined with that of hydrogen sulfide, are given below.

## 2. Hydrogen Sulfide Metabolism in Human Physiology

The reactivity and potential toxicity of hydrogen sulfide demand its levels to be kept under strict control. Indeed, even temporary imbalances in local hydrogen sulfide concentrations can trigger a cascade of cellular events with pathological consequences. The production and breakdown of H_2_S is thus essentially ensured by specialized enzymes, tightly regulated and compartmentalized. Although H_2_S is freely permeable to membranes and thus highly diffusible inside the cell, compartmentalization of its synthesis or breakdown could be relevant to ensure local effects in cellular organelles. A clue for this has been for instance provided by showing that the mitochondria-targeted H_2_S donor AP39 ([10-oxo-10-(4-(3-thioxo-3*H*-1,2-dithiol-5yl)phenoxy)decyl) triphenylphosphonium bromide]), consisting of the H_2_S-releasing moiety ADT-OH (5-(4-hydroxyphenyl)-3*H*-1,2-dithiole-3-thione)coupled to the mitochondria-targeting triphenylphosphonium (TPP^+^) moiety, protects mitochondria in oxidatively stressed endothelial cells more effectively than ADT-OH itself, not coupled to TPP^+^ and thus unable to specifically target mitochondria [7]. Despite decades of molecular and cellular studies on the enzymatic systems involved in H_2_S synthesis and breakdown, it appears at times that this field of biology is still in its infancy, with new reactive molecules and new targets of H_2_S and related species being consistently identified and new mechanistic details and regulatory subtleties being unraveled.

### 2.1. Human H_2_S-Synthesizing Enzymes

The biogenesis of hydrogen sulfide in human physiology occurs via two major routes: by endogenous specialized enzymes and as an end-product or intermediate of microbial metabolic pathways within the gut microbiota, particularly in sulfate-reducing bacteria. Interestingly, by comparing germ-free versus conventional mice, it was shown that the intestinal microbiota regulates H_2_S homeostasis not only in the gut but also systemically in various tissues and organs [8]. Namely, the presence of microbiota was found to be associated with higher levels of free H_2_S not only in some intestinal tracts (colon and cecum) but also in plasma. Moreover, as compared to germ-free mice, the conventional animals displayed higher levels of bound sulfane sulfur in plasma, fat, and lung, and higher CSE activity and reduced cysteine levels in most organs and tissues [8]. Another secondary source of H_2_S is proposed to be persulfides and polysulfides, either endogenously generated or derived from dietary intake (reviewed in, e.g., [9]).

**Figure 1:**
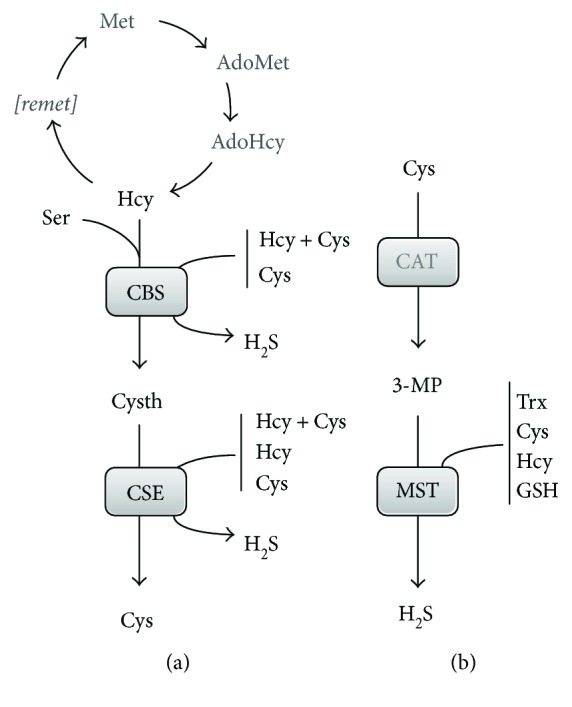
Hydrogen sulfide biosynthetic pathways in mammalian physiology. (a) Transsulfuration branch of the methionine cycle. *l*-Methionine (Met) is converted by *l*-methionine adenosyltransferase to *s*-adenosyl-*l*-methionine (AdoMet), which is used by methyltransferases in methylation reactions, generating *s*-adenosyl-*l*-homocysteine (AdoHcy). AdoHcy hydrolase then converts AdoHcy into *l*-homocysteine (Hcy), which can either be converted back to *l*-methionine through the remethylation cycle ([remet]), or enter the transsulfuration branch. Cystathionine *β*-synthase (CBS) converts Hcy and *l*-serine into *l*-cystathionine (Cysth), which is taken up by cystathionine *γ*-lyase (CSE) to generate *l*-cysteine (Cys). Hydrogen sulfide (H_2_S) is synthesized by CBS and CSE in several alternative reactions (see Figure 2). (b) H_2_S synthesis by 3-mercaptopyruvate sulfurtransferase (MST). Cys is converted by cysteine aminotransferase (CAT) into 3-mercaptopyruvate (3-MP), which is used by MST to synthesize H_2_S along with its cosubstrates thioredoxin (Trx), Cys, Hcy, and *l*-glutathione (GSH).

The three human enzymes reported to endogenously generate hydrogen sulfide are cystathionine *β*-synthase (CBS), cystathionine *γ*-lyase (CSE, also known as cystathionase), and 3-mercaptopyruvate sulfurtransferase (MST) (Figure 1) [10].

#### 2.1.1. Expression in Cells and Tissues and Cellular Localization

**Figure 2:**
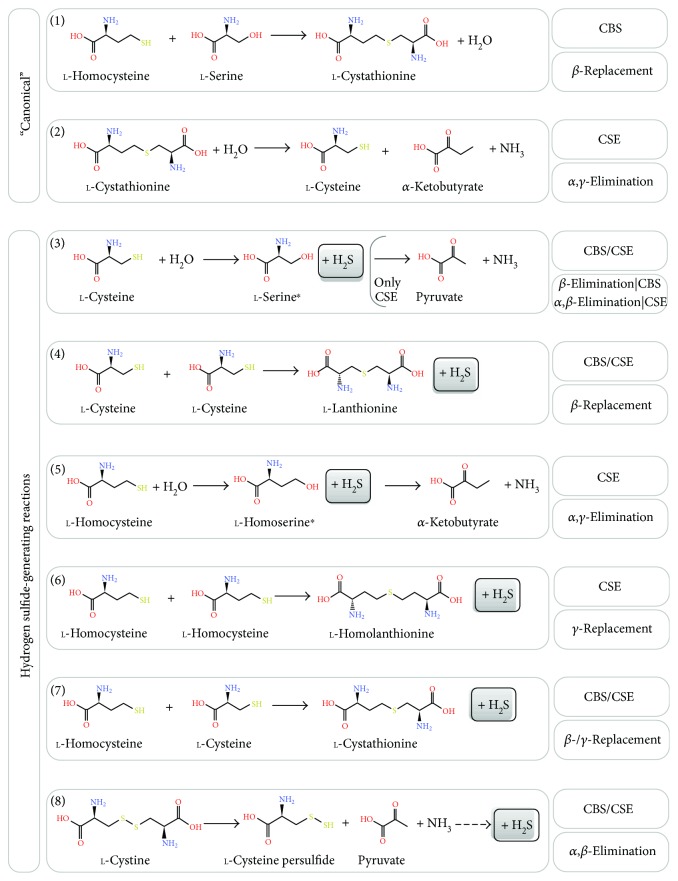
Catalytic versatility of the H_2_S-synthesizing enzymes cystathionine *β*-synthase (CBS) and cystathionine *γ*-lyase (CSE). Chemical structure of the metabolites and reaction schemes involved in H_2_S synthesis by CBS and CSE. Top panel, “canonical” reactions catalyzed by CBS and CSE as part of the transsulfuration branch of the methionine cycle. Center-bottom panel, alternative H_2_S-generating reactions. Chemical structures labeled as *α*-ketobutyrate and pyruvate are represented as the *α*-ketobutyric and pyruvic acid, respectively. ^∗^Serine and homoserine have not been detected as intermediates in the CSE-catalyzed reactions 3 and 5, respectively, only their downstream products *α*-ketobutyric and pyruvic acids (resp.).

The occurrence of all three H_2_S-synthesizing enzymes in cells, tissues, and organs and their cellular distribution remain evolving subjects, particularly taking into account that these may vary under different (patho)physiologic conditions. It is generally considered that CBS regulation occurs at the protein level, the enzyme being a target of multiple posttranslational modifications and having two regulatory domains that respond to different stimuli (reviewed, e.g., in [11]), unlike CSE, for which no posttranslational regulatory mechanisms are known [10]. Moreover, since CBS and CSE employ various combinations of the same substrates in their H_2_S-generating reactions (Figures 1 and 2), regulation of one enzyme will likely affect the other, due to increased or decreased substrate availability (e.g., in [11]).

The tissue and organ distribution of the H_2_S-synthesizing enzymes has been studied for different organisms, employing immunohistochemical detection and analyzing transcriptional levels and H_2_S-generating activity. In man and rodents, CBS is mostly abundant in the liver, pancreas, kidney, brain, and nervous system [9, 12–14]. Recently, CBS expression in the carotid body, as well as in uterine and mesenteric and umbilical arteries, was reported [15]. As for CSE, it is mostly expressed in the liver, kidney, and smooth muscle (both vascular and nonvascular), its expression and H_2_S-generating activity being negligible in the brain, heart, and spleen [9, 13, 16, 17]. MST tissue distribution has been systematically studied for different organisms. The activity of bovine MST has been reported to be highest in the adrenal cortex, followed by the liver, heart, and kidney [18]. Abundant rat MST expression has been detected in the kidney (proximal tubular epithelium), liver (pericentral hepatocytes), aorta, and brain glial cells [19]. Recently, Tomita et al. detected abundant murine MST in the brain (neural and glial cells), liver, kidney, testes, and endocrine organs (particularly in pancreatic islets) and lower levels in bronchiolar cells, spleen, thymus, and small intestine [20]. Finally, Western blot analysis and enzymatic assays revealed the presence of active MST, but undetectable CBS and CSE, in red blood cells [21].

In terms of cellular localization, the classical view is that both CBS and CSE are cytosolic enzymes, whereas MST localizes to the mitochondria (e.g., [9, 10, 22]). However, several examples show that this classical view may be challenged. It has been reported that when blood homocysteine levels rise, microvascular endothelial cells and hepatocytes may secrete CBS and CSE, thus circulating as part of the plasma proteome and contributing to H_2_S generation [23]. Extracellularly synthesized H_2_S was shown to contribute to increased cell viability and decreased oxidative damage to DNA after serum starvation and hypoxia/reoxygenation. Furthermore, immunoprecipitation of circulating CBS and CSE from serum prior to its supplementation with homocysteine enhanced serum-induced stress towards endothelial cells. Altogether, these observations suggest that secreted CBS and CSE have a protective role in the endothelium by both producing H_2_S and clearing excess homocysteine. Another notable characteristic of CBS is its ability to be SUMOylated leading to protein accumulation in the nucleus [24]. Perhaps of greatest significance for their role as H_2_S-synthesizing enzymes is the observation that both CBS and CSE can translocate to the mitochondria under various (patho)physiological conditions. This becomes particularly relevant taking into account that H_2_S can not only stimulate mitochondrial bioenergetics (i) by supplying electron equivalents to the quinol pool via sulfide:quinone oxidoreductase (reviewed in [10, 25]), (ii) by activating the glycolytic enzyme glyceraldehyde 3-phosphate dehydrogenase [26], and (iii) by persulfidation of ATP synthase [27, 28] but also block mitochondrial respiration by tight inhibition of cytochrome *c* oxidase (CcOX, reviewed in [29–31]). CBS has been observed to partially localize to liver mitochondria, where the protein transiently accumulates under hypoxia/ischemia, with the resulting increased H_2_S generation protecting mitochondria from oxidative stress [32]. Once cells return to normoxia, CBS levels are restored via protein degradation by mitochondrial Lon protease, which recognizes and targets specifically the oxidized form of the regulatory heme at the CBS N-terminal domain [32].

In the colorectal cancer cell line HCT 116, a significant fraction of CBS localizes to the mitochondria, being associated with the outer mitochondrial membrane. The resulting increased H_2_S levels are proposed to stimulate cancer cell energy metabolism, particularly oxidative phosphorylation and glycolysis [33]. In line with this observation, CBS has been shown to contribute in various cancer cells to a shift in the fate of glucose utilization from glycolysis to the pentose phosphate pathway [34] (detailed in Section 4.3.1). By also promoting tumor angiogenesis, the increased H_2_S availability may contribute to increased oxygen supply to tumor cells. In a mouse model of amyotrophic lateral sclerosis, CBS accumulation in mitochondria isolated from the spinal cord and the resulting increased H_2_S generation are proposed to be related with impaired cytochrome *c* oxidase-dependent respiration [35].

CSE has been reported to translocate to the mitochondria of vascular smooth muscle cells (SMCs) in response to stimuli that increased intracellular calcium levels. This translocation appears to be mediated by the mitochondrial membrane translocase of the outer membrane 20 (Tom20). Whereas SMCs display impaired ATP production in hypoxic conditions, the presence of CSE inside the mitochondria contributed to cysteine catabolism, H_2_S synthesis, and stimulated ATP synthesis under such conditions, suggesting that CSE translocation may sustain mitochondrial bioenergetics in response to certain stresses [28].

MST is generally regarded as a mitochondrial enzyme. However, Fräsdorf et al. have shown that there are actually two splice variants of the protein, MST-Iso1 and MST-Iso2, both of which localize to the cytosol, while only MST-Iso2 also localizes to the mitochondria in HEK293a and HeLa cells [36].

#### 2.1.2. Associated Metabolic Pathways

CBS and CSE participate in the transsulfuration branch of the methionine methylation/remethylation cycle (Figure 1(a)), a key pathway which generates extremely relevant signaling molecules for mammalian physiology. In this cycle, methionine (Met) derived essentially from dietary intake is converted to *s*-adenosyl-*l*-methionine (AdoMet) by methionine adenosyltransferase ([37] and references therein). AdoMet is the methyl group donor for virtually all methylation reactions in mammalian physiology, through the action of methyltransferases. Concurrently with methylation, *s*-adenosyl-*l*-homocysteine (AdoHcy) is generated, which is used by AdoHcy hydrolase (SAHH) to produce homocysteine (Hcy) and adenosine. Homocysteine then either enters the transsulfuration branch or proceeds through the remethylation pathway to revert to methionine, depending on different metabolic fluxes and other physiological conditions. The metabolites AdoMet, AdoHcy, and Hcy participate in the regulation of numerous physiological processes. As detailed below, AdoMet is an allosteric regulator of CBS, increasing its enzymatic activity by 2–5-fold upon binding. This allosteric activation results in an increase in *V*_max_, without any notable effect on the substrate affinity (*K*_*M*_) [38, 39]. The “canonical” reactions attributed to CBS and CSE within the transsulfuration pathway result in conversion of homocysteine into cysteine as follows (Figure 2): CBS catalyzes the *β*-replacement of serine by homocysteine, yielding cystathionine, and CSE then proceeds by converting cystathionine to cysteine, *α*-ketobutyrate, and ammonia in an *α*,*γ*-elimination reaction. The role of CBS and CSE in hydrogen sulfide production results from an extensive repertoire of alternative reactions that these enzymes are able to catalyze (Figure 2) [39, 40], possibly related to the fact that both are pyridoxal 5′-phosphate- (PLP-) dependent enzymes. This may be perceived as catalytic promiscuity or versatility-based robustness [11]. A notable thorough *in vitro* kinetic characterization of CBS- and CSE-catalyzed H_2_S-generating reactions combined with kinetic simulations has been carried out by Singh et al., allowing to envisage which reactions are relevant *in vivo* under different (patho)physiological conditions [39]. Both CBS and CSE are able to synthesize H_2_S through cysteine *β*-elimination or *β*-replacement, respectively employing one or two cysteine molecules (Figure 2, reactions (3) and (4)), or through *β*- or *γ*-replacement using cysteine and homocysteine (Figure 2, reaction (7)). CSE alone is able to synthesize H_2_S via homocysteine *α*,*γ*-elimination or *γ*-replacement, respectively employing one or two homocysteine molecules (Figure 2, reactions (5) and (6)). Both CBS and CSE also catalyze cystine *α*,*β*-elimination to yield cysteine persulfide, which ultimately may generate H_2_S (Figure 2, reaction (8)). Besides H_2_S synthesis, some of these reactions originate thioether metabolites such as lanthionine and homolanthionine, which may have been suggested as biomarkers of hydrogen sulfide generation in homocystinuric patient samples [41]. Under substrate saturating conditions, the turnover number of recombinant human CBS for the H_2_S-generating cysteine and homocysteine *β*-/*γ*-replacement reaction (reaction (7) in Figure 2) is 3.7-fold higher than that of the cystathionine-generating canonical reaction [39]. Kinetic simulations show that this difference drops to 1.3-fold at physiologically relevant substrate concentrations, which still indicates that CBS-catalyzed H_2_S synthesis at the expense of homocysteine and cysteine accounts for ~56% of cystathionine production. Notably, reaction (7) is responsible for 96% of the net CBS-catalyzed H_2_S production, whereas the cysteine-alone-based reactions (reactions (3) and (4) in Figure 2) account for a mere 1.6–2.6% of H_2_S synthesis. Still, the H_2_S-producing capacity of CBS appears to be insensitive to the systemic homocysteine levels, possibly related to the fact that homocysteine can only bind to CBS after serine or cysteine. The same kinetic simulations show that, contrarily to CBS, CSE catalyzes the canonical reaction of cysteine synthesis via cystathionine *α*,*γ*-elimination (reaction (2) in Figure 2) with a much higher (20–30 fold) catalytic efficiency (*k*_cat_/*K*_*M*_) than determined for the H_2_S-synthesizing reactions (reactions (3–6) in Figure 2). At physiologically relevant substrate concentrations (10 *μ*M homocysteine, 100 *μ*M cysteine, and 5 *μ*M cystathionine), the canonical reaction turnover is still 5-fold or 12-fold higher than those for H_2_S-generating cysteine or homocysteine cleavage reactions, respectively. Under those conditions, approximately 70% of CSE-catalyzed H_2_S production derives from cysteine and 29% from homocysteine. Also in contrast with CBS, the CSE-catalyzed H_2_S generation depends on homocysteine availability. Indeed, kinetic simulations assuming equimolar CBS (fully activated by AdoMet) and CSE at “normal” homocysteine concentrations (10 *μ*M) reveal that CSE contributes to 32% of the synthesized H_2_S, whereas the CSE contribution increases to 45% and 74% under conditions of moderate and severe hyperhomocysteinemia, respectively. This indicates that CSE may have a significant role in homocysteine clearance at pathophysiological elevated homocysteine levels, particularly in tissues or organs where CBS is absent or poorly expressed.

Recently, Majtan et al. reported on a thorough kinetic analysis of CBS-catalyzed reactions combined with simulations [38]. This study demonstrated the competitive advantage of serine over cysteine as a substrate for its condensation with homocysteine (to yield H_2_O or H_2_S, resp.), with a 2–5-fold higher catalytic efficiency of the canonical reaction compared to the H_2_S-generating reaction. However, despite this apparent advantage at saturating substrate concentrations, the H_2_S-producing reaction is able to compete with the canonical reaction under physiologically relevant conditions [38]. Indeed, one of the key findings is that a main determinant of CBS-catalyzed H_2_S production is the cysteine to serine ratio, adding another layer of complexity to be taken into account when considering the *in vivo* role of each H_2_S-producing enzyme.

Despite the remarkable value of these kinetic studies, H_2_S generation obviously also depends on the relative expression levels of each enzyme in each cell/tissue and on the systemic and local availability of substrates (cysteine, homocysteine, cystine, and serine), regulatory metabolites (e.g., AdoMet), and other effectors which negatively modulate enzymatic activity (e.g., NO and CO inhibit CBS; detailed below).

**Figure 3:**
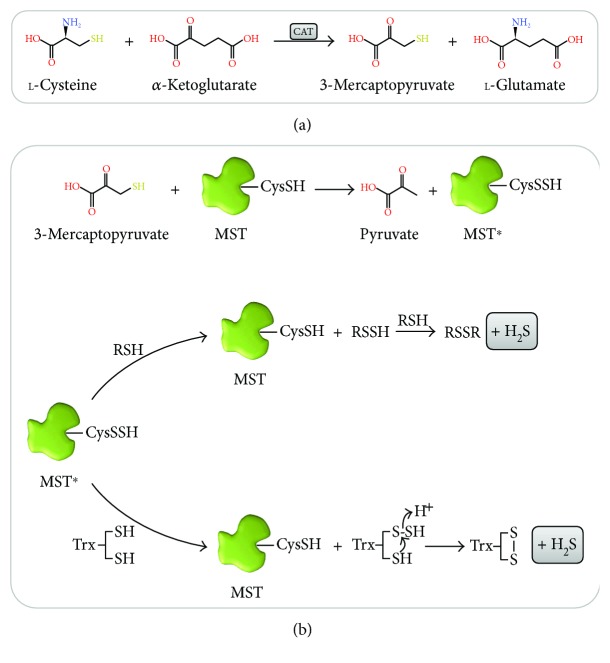
Reaction mechanism of H_2_S production by 3-mercaptopyruvate sulfurtransferase (MST). Chemical structure of the metabolites and reaction schemes involved in H_2_S synthesis by MST. (a) Production of 3-mercaptopyruvate (3-MP) is catalyzed by cysteine aminotransferase (CAT), with *α*-ketoglutarate as cosubstrate. (b) MST reaction with its activating substrate 3-MP and acceptor substrates. Upon reacting with 3-MP, the side-chain sulfhydryl of Cys_248_ (CysSH) becomes persulfidated (CysSSH), which activates MST (MST^∗^) towards the reaction with the acceptor substrates. The reaction with low-molecular-weight (LMW) thiol-containing substrates (RSH) involves the sequential reaction with two substrate molecules. In the first step, MST^∗^ transfers the sulfane sulfur from the cysteine persulfide to the RSH, regenerating the enzyme and releasing a persulfide RSSH product. In the second step, another RSH reacts with RSSH to yield H_2_S and oxidized RSSR. The reaction of MST^∗^ with reduced thioredoxin (Trx) proceeds similarly to that with LMW RSH molecules. The MST cysteine persulfide is initially transferred to Trx, yielding CysSSH, which is then attacked by a nearby cysteine thiol, releasing H_2_S and yielding oxidized Trx. Chemical structures labeled as *α*-ketoglutarate, 3-mercaptopyruvate, and *l*-glutamate are represented as *α*-ketoglutaric, 3-mercaptopyruvic, and *l*-glutamic acids, respectively.

The role of 3-mercaptopyruvate sulfurtransferase (MST) is also tightly associated with cysteine metabolism. 3-Mercaptopyruvate (3-MP) is generated by cysteine (or aspartate) aminotransferase (CAT, Figure 3(a)) through cysteine deamination, using *α*-ketoglutarate as cosubstrate and yielding glutamate as coproduct [10]. An additional pathway whereby 3-MP is generated has been recently demonstrated to be present mostly in the cerebellum and the kidney, involving D-cysteine and a D-amino acid oxidase [42].

The interaction between MST and its 3-MP substrate gives rise to an enzyme-bound cysteine persulfide intermediate at Cys_248_ (MST^∗^ in Figure 3(b)), concomitantly with pyruvate release [43]. H_2_S can then be released by transfer of the active site cysteine persulfide to physiologic small thiols like cysteine, homocysteine, and glutathione or reducing molecules such as thioredoxin (Trx) or dihydrolipoic acid (DHLA) or by reaction with nonphysiological reductants like 2-mercaptoethanol and dithiothreitol [43]. The MST persulfide intermediate transfers its persulfide to an acceptor R-SH, forming an R-S-SH intermediate, which then reacts with another acceptor RSH molecule to yield a disulfide (RSSR) and H_2_S. From the tested physiological acceptor substrates, thioredoxin displays the highest catalytic efficiency, orders of magnitude higher than those for DHLA, cysteine, homocysteine, and glutathione [43]. This observation is consistent with the observation that some protozoan parasites encode MST variants with a fused thioredoxin domain.

The broad impact of H_2_S on mammalian physiology has been widely assessed in numerous *in vivo* studies, utilizing knockout mouse models of the three H_2_S-synthesizing enzymes CBS, CSE, and MST and of ethylmalonic encephalopathy protein 1 (ETHE1, also known as persulfide dioxygenase or sulfur dioxygenase), implicated in H_2_S breakdown. Making use of these models, particularly the ones for CSE and CBS, H_2_S has been shown to take part in a huge number of physiological and pathophysiological processes and thus to have a high impact on human health and disease. For a comprehensive overview of the wide spectrum of studies carried out on these models, the readers can refer to [9], a recent review specifically addressing this topic.

#### 2.1.3. Structural and Functional Properties of H_2_S-Generating Enzymes

**Figure 4:**
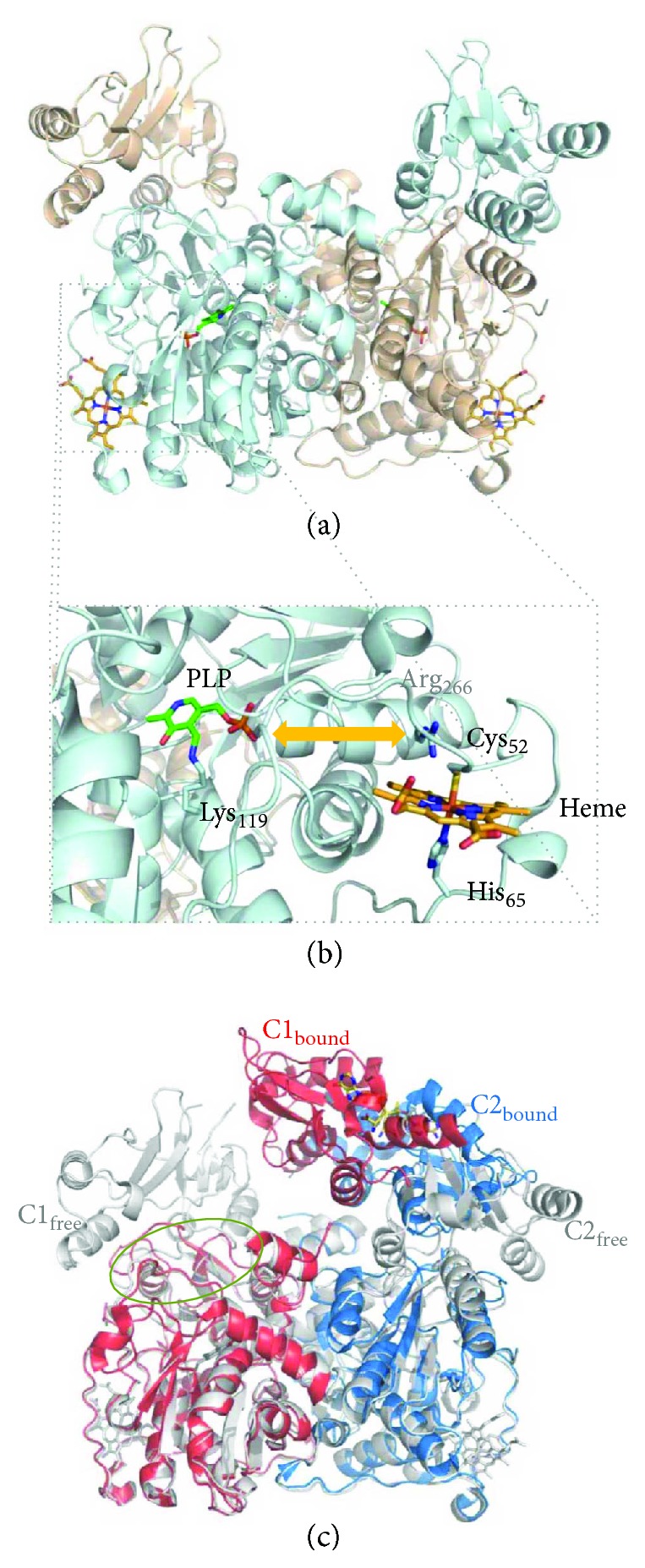
Crystallographic structure of human cystathionine *β*-synthase (CBS). (a) Cartoon representation of the “full-length” human CBS homodimer (PDB ID: 4COO; *Δ*516–525; 2.0 Å resolution). Green sticks, active site PLP moiety, where H_2_S production occurs. Orange sticks, regulatory heme *b* where CO or NO binds, resulting in enzyme inhibition. (b) Zoom-in into the catalytic (PLP) and regulatory (heme) sites. The PLP moiety is covalently attached to CBS through Lys_119_ (human CBS numbering), while the *b*-type heme is axially coordinated by Cys_52_ and His_65_. Orange arrow indicates the communication between the heme and PLP sites mediated by the *α*-helix comprising residues Thr_257_-Gly_258_-Gly_259_-Thr_260_-Ile_261_-Thr_262_-Gly_263_-Ile_264_-Ala_265_-Arg_266_. (c) Structural effect of AdoMet binding. While in AdoMet-free CBS, the C-terminal domain of each monomer (*C*1_free_ and *C*2_free_, colored in light gray) blocks the substrate entrance into the active site (green oval circle) of the adjacent monomer, AdoMet binding to the C-terminal domains leads to association of the latter in a disk-like form (*C*1_bound_ and *C*2_bound_; PDB ID: 4PCU; Δ516–525 Glu201Ser; 3.58 Å resolution), unblocking the active site and derepressing the enzymatic activity. Figure generated with PyMol 1.8.2.0 [238].

*(1) Cystathionine β-Synthase (CBS)*. Human cystathionine *β*-synthase (CBS) is considered to assemble as a homotetramer of 551 amino acid-long (~61 kDa) monomers, each consisting of three domains (Figure 4(a)): an N-terminal domain (residues 1–70) that binds a noncatalytic heme cofactor, a central catalytic PLP-binding domain (residues 71–381), and a C-terminal regulatory domain (residues 412–551, also known as Bateman module, comprising two motifs, CBS1 and CBS2) responsible for enzyme activation upon AdoMet binding. Structural studies on the human enzyme have revealed that the catalytic domain has a highly conserved structural fold of the *β*-family of PLP-dependent enzymes, consisting of thirteen *α*-helices and two *β*-sheets composed of four and six strands [44, 45]. Several intrinsically flexible regions that were initially not visible in the structure of a truncated human CBS lacking the C-terminal domain became visible in the structure of a “full-length” CBS construct (with a 10-residue deletion at the C-terminus, (Figure 4(a)). These include the strands *β*4, *β*5, and *β*6 preceding the loops L145–148, L171–174, and L191–202, which are proposed to constitute the catalytic site entrance (together with loop L295–316) and are stacked between the catalytic core and the C-terminal domain, as well as helix *α*7 following the L191–202 loop. The active site PLP moiety is bound to the catalytic core through a Schiff base bond to the *ε*-amino group of lysine 119 (Figure 4(b)). A long 30-residue *α*-helical stretch (residues 382–411; helices *α*15 and *α*16) tethers the catalytic core to the C-terminal AdoMet-binding domain. The latter actually comprises two so-called CBS domains which share a common structural fold despite the poor sequence identity (~7% over 133 residues). The key finding in the structure of full-length CBS was the revelation that, in AdoMet-free CBS, the C-terminal domain from one monomer blocks the substrate entry site of the opposing monomer within one dimer (Figure 4(c)) [44]. This blockage is accomplished by stabilization of the above mentioned intrinsically flexible region composed by the *β*4, *β*5, and *β*6 strands preceding the loops L145–148, L171–174, and L191–202. Indeed, the interaction between the core and regulatory domains has been shown to involve (i) hydrophobic interactions with residues from the C-terminal domain CBS2 motif and (ii) H-bonds with residues from the C-terminal domain CBS1 motif [44, 46]. Notably, in the presence of AdoMet, the two C-terminal domains assemble together in a “disc”-like form, similarly to the constitutively activated *Drosophila melanogaster* AdoMet-free CBS [47], the substrate entrance opens, and access to the active site is enhanced (Figure 4(c)), thus increasing enzymatic activity [44, 46, 48]. Each Bateman module contains two putative AdoMet-binding cavities (S1 and S2) with different binding affinities [49]. One of these cavities (S2) is exposed and thus likely represents the primary AdoMet binding site, while the other one (S1) appears to be hindered by bulky hydrophobic residues from the catalytic core [46, 48, 49]. The AdoMet-induced association between adjoining Bateman modules disrupts the interactions between the catalytic and C-terminal regulatory domains and opens the catalytic core to the substrates.

**Figure 5:**
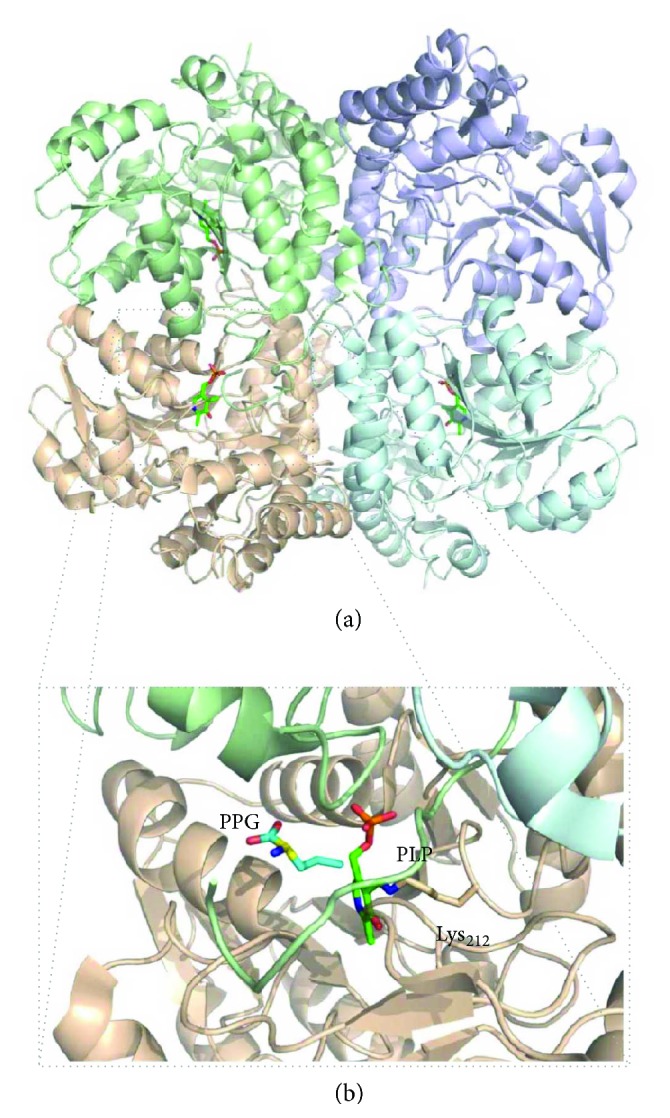
Crystallographic structure of human cystathionine *γ*-lyase (CSE). (a) Cartoon representation of human CSE homotetramer (PDB ID: 3COG; 2.0 Å resolution) cocrystallized with the inhibitor propargylglycine (PPG). Each chain is represented in a different colour. Green sticks, active site PLP moiety where H_2_S production occurs. (b) Zoom-in into the catalytic PLP site. The PLP moiety (green sticks) is covalently attached to CSE through Lys_212_ (human CSE numbering); PPG is represented in blue sticks. Figure generated with PyMol 1.8.2.0 [238].

*(2) Cystathionine γ-Lyase (CSE)*. Cystathionine *γ*-lyase (CSE) is a homotetrameric enzyme constituted by 405-amino-acid-long ~44 kDa monomers, each consisting of two structural domains (Figure 5(a)) [50]. The larger PLP-binding catalytic domain (comprising residues 9–263) assembles as an *α*/*β*/*α* fold consisting of a seven-stranded *β*-sheet (6 parallel and 1 antiparallel) flanked by eight *α*-helices. The smaller C-terminal domain (residues 264–401) is composed of a *β*-sheet (4 antiparallel strands) with three helices on one side. Similarly to CBS, the PLP in CSE is anchored through a Schiff base bond between the PLP carbonyl and the *ε*-amino group of a lysine residue (Lys_212_) (Figure 5(b)), although other forces are at play to stabilize PLP: *π*-stacking interactions between the Tyr_114_ phenyl moiety and the PLP pyridine ring, and H-bonds between the PLP phosphate moiety and residues Gly_90_, Leu_91_, Ser_209_, and Thr_211_ from the same subunit and Tyr_60_ and Arg_62_ from the adjacent subunit. Structural studies on CSE in the presence of its inhibitor propargylglycine (PPG) attempted to explain its mode of action [50]. While the lysine-PLP bond appears to remain unaffected, PPG is proposed to covalently bind to Tyr_114_, becoming a vinyl ether, while also forming H-bonds between its amino group and Glu_339_ and between its carboxyl moiety and Arg_119_ and Arg_62_ from the other monomer. This static position of the covalently bound PPG vinyl ether is such that it extends towards the internal PLP aldimine, thus blocking the cofactor reactivity [50].

**Figure 6:**
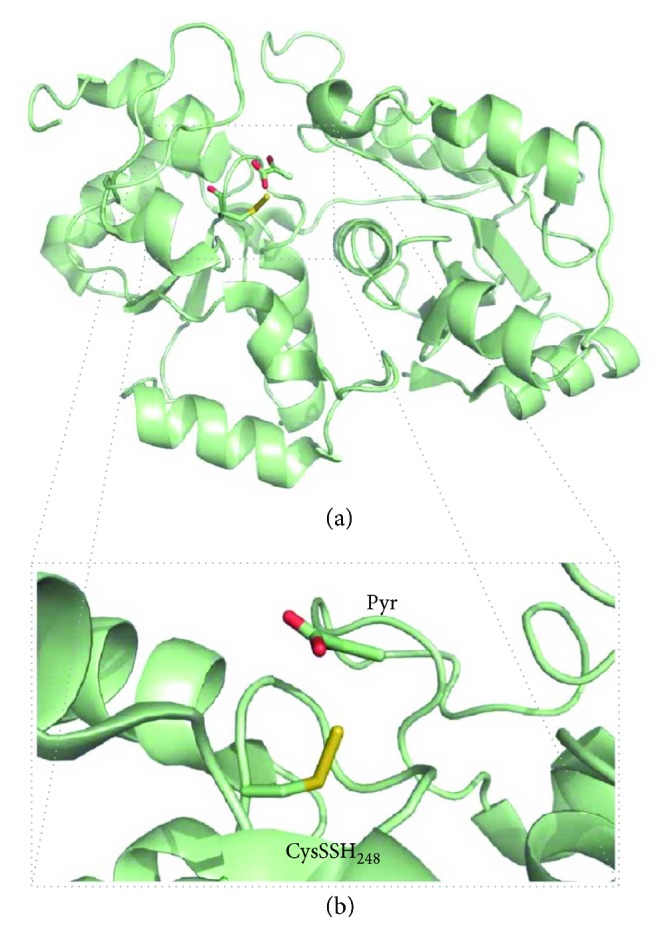
Crystallographic structure of human 3-mercaptopyruvate sulfurtransferase (MST). (a) Cartoon representation of human MST monomer (PDB ID: 4JGT; 2.16 Å resolution) cocrystallized with its substrate 3-mercaptopyruvate (3-MP). (b) Zoom-in into the catalytic persulfidated Cys_248_ (CysSSH_248_; human MST numbering) site, after reaction with 3-MP. Pyruvate, a by-product of the reaction of MST with 3-MP, is represented in green sticks. Figure generated with PyMol 1.8.2.0 [238].

*(3) Mercaptopyruvate Sulfurtransferase (MST)*. Human mercaptopyruvate sulfurtransferase (MST) is composed of 297 amino acid residues and assembles as a ~33 kDa monomer consisting of two structurally related domains with a rhodanese-like fold (Figure 6(a)) [43]. The N-terminal (residues 1–138) and C-terminal (residues 165–285) domains are tethered by a 26-amino acid linker that strongly interacts with both domains. The assembly of these structurally related domains could have arisen from gene duplication. Structural studies of MST in the presence of its 3-MP substrate have shown that it binds to cysteine 248 in the active site, which is located in a cleft between the two domains (Figure 6(b)) [43]. The reaction originates a persulfidated CysSSH-activated intermediate. Despite the significant substrate-induced chemical modification of the active site, a comparison between the structures of MST with and without bound 3-MP reveals that no major structural differences occur [43]. In a recent report on a compound screening campaign targeting MST, the structures of MST in complex with hit compounds revealed a strong interaction between the persulfidated Cys_248_ and the inhibitors' pyrimidone-like aromatic rings [51], without major structural changes.

### 2.2. H_2_S Catabolism

#### 2.2.1. H_2_S: A “Self-Regulatory” Janus Molecule for Cellular Bioenergetics

**Figure 7:**
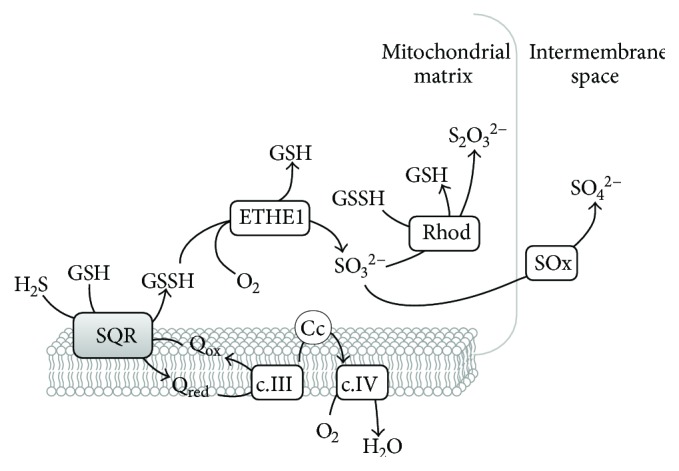
Mitochondrial sulfide-oxidizing pathway. Scheme depicting the enzymatic components and metabolites involved in sulfide oxidation in the mitochondria. H_2_S is initially oxidized by sulfide:quinone oxidoreductase (SQR), which transfers electron equivalents to quinones and generates glutathione persulfide (GSSH) as coproduct. Electrons are transferred to O_2_ via complex III (c.III), cytochrome *c* (Cc), and complex IV (c.IV), contributing to membrane energization and ATP synthesis. GSSH, with O_2_ as cosubstrate, is then converted by persulfide dioxygenase (ETHE1) to sulfite (SO_3_^2−^) and GSH. Sulfite can be converted, with GSSH as cosubstrate, into thiosulfate (S_2_O_3_^2−^) by rhodanese (Rhod), or oxidized into sulfate (SO_4_^2−^) by sulfite oxidase (SOx).

As mentioned above, while playing a key signaling role only at relatively low, physiological concentrations, at higher concentrations H_2_S is potentially toxic, being able to impair cell respiration through inhibition of CcOX [52]. To prevent toxicity, H_2_S bioavailability must therefore be finely and differently regulated in different tissues and organs, depending on specific physiological demands. Control of H_2_S bioavailability is exerted not only at the level of its biosynthesis but also through enzymatic disposal of such a potentially toxic molecule. In mammals, the ability to detoxify H_2_S to thiosulfate and sulfate was demonstrated by metabolic labelling in early studies [53]. More recently, it was discovered that H_2_S breakdown is mainly afforded by a mitochondrial enzymatic system (reviewed in [10, 25]), currently designated as “sulfide-oxidizing unit” [54] or “sulfide-oxidizing pathway”. Following its initial identification in the lugworm *Arenicola marina* [55], this mitochondrial system has been investigated in more detail. According to current views, this pathway comprises four distinct, yet functionally associated enzymes that together cooperate to catalyze the breakdown of H_2_S to thiosulfate and sulfate, the main sulfide catabolites: a sulfide:quinone oxidoreductase (SQR), a persulfide dioxygenase (also known as ETHE1 or sulfur dioxygenase), a thiosulfate sulfurtransferase (rhodanese), and a sulfite oxidase (SO*_x_*) (Figure 7). It is to be noted that H_2_S oxidation to polysulfide can also be catalyzed by globins (see below) and other proteins, such as catalase and superoxide dismutase, utilizing O_2_ or H_2_O_2_ as electron acceptor [56–59].

From several perspectives, it is fascinating that H_2_S oxidation by mitochondria is coupled to energy production, namely, ATP synthesis, as initially demonstrated by investigating the invertebrate *Solemya reidi* [60]. Electrons derived from sulfide oxidation are indeed injected into the respiratory chain at the level of coenzyme Q, thus promoting O_2_ consumption and, in turn, energization of the inner mitochondrial membrane. On this basis, H_2_S has been recognized as the first inorganic respiratory substrate discovered in mammals [61]. This makes H_2_S a very peculiar molecule from a bioenergetic point of view [62], with a dual effect on mitochondrial respiration: stimulatory at low (nanomolar) concentrations when the mitochondrial sulfide oxidation pathway is fully operative or inhibitory at higher (micromolar) concentrations when CcOX activity is impaired and reduced coenzyme Q accumulates, leading to blockage of H_2_S detoxification. As remarked recently [63], the system is therefore finely regulated through positive feedback loops so that at lower concentrations sulfide oxidation prevents its own accumulation and consequent inhibition of respiration, whereas at higher concentrations sulfide impairs its own detoxification via inhibition of CcOX. That H_2_S can act as an effective substrate of the mitochondrial respiratory chain is in full agreement with the observation that, despite the low *K*_*i*_ value measured with isolated CcOX (*K*_*i*_ = 0.2 *μ*M at pH 7.4 [52]), in isolated mitochondria or intact cells, inhibition of respiration only occurs if much higher concentrations of H_2_S are administered (micromolar to tens of micromolar; see, for instance, [64, 65]).

The dual effect of H_2_S on cell respiration makes investigation of the mitochondrial sulfide-oxidizing activity somewhat challenging from a methodological point of view. Sulfide stimulation of mitochondrial oxygen consumption and consequent energization are indeed best appreciated at low sulfide concentrations, unable to inhibit CcOX. For this reason, most of studies on mitochondrial sulfide oxidation and related bioenergetic effects have been carried out by continuous supply of sulfide at given infusion rates, rather than by sulfide addition as a single bolus (method reviewed in [66]). Using this approach, sulfide oxidation has been quantitatively investigated in several systems, including isolated mitochondria and permeabilized or intact cells from humans and other higher organisms [54, 61, 63, 67–72]. Notably, remarkable differences in terms of H_2_S oxidation ability have been reported between human cell lines, spanning from cells in which this catalytic activity is undetectable (as, e.g., in neural cells) to others remarkably active in H_2_S removal, like colonocytes [54]. The latter cells are indeed physiologically exposed to massive amounts of H_2_S produced by the intestinal microbiota and are therefore likely to have undergone adaptive mechanisms to prevent H_2_S toxicity. In this regard, it is interesting that colonocytes were found to ensure a prominent H_2_S-catabolyzing activity even through activity reversal of complex I in the respiratory chain [54].

#### 2.2.2. Components of the Mitochondrial Sulfide-Oxidizing Pathway

**Figure 8:**
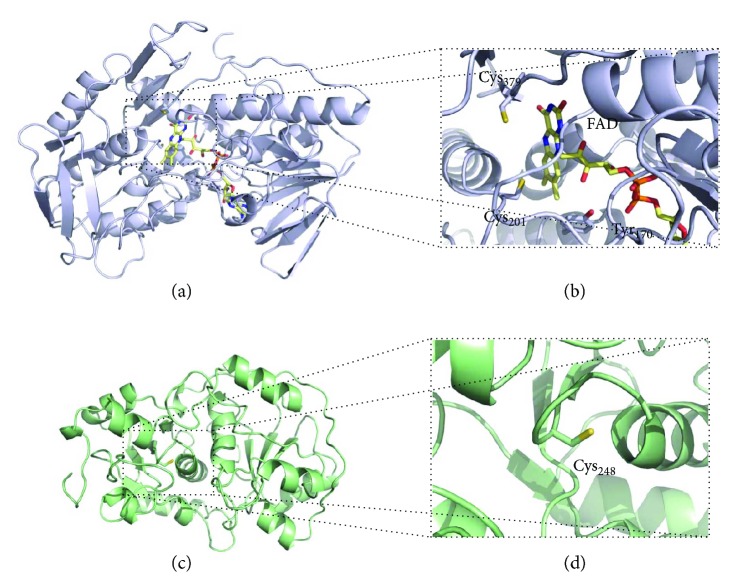
Structural models of human sulfide quinone oxidoreductase (SQR) and rhodanese (Rhod). (a) Cartoon representation of structural model of human SQR (UniProt accession code: Q9Y6N5.1) generated with Swiss-Model based on the structure of *Acidithiobacillus ferrooxidans* SQR (H198A variant; PDB code: 3SZF; ~19% sequence identity; ~87% sequence coverage) [78]. Flavin adenine dinucleotide (FAD) cofactor in yellow sticks. (b) Zoom-in on the SQR active site comprising the FAD moiety, the active site cysteine residues Cys_201_ and Cys_379_, and the Tyr_170_ residue which may establish a covalent link with the FAD cofactor. (c) Cartoon representation of structural model of human rhodanese (UniProt accession code: Q16762.4) generated with Swiss-Model based on the structure of the bovine enzyme (PDB code: 1BOH; ~90% sequence identity; 100% sequence coverage). (d) Zoom-in on the Rhod Cys_248_ active site. Figure generated with PyMol 1.8.2.0 [238].

The first enzyme operating in the mitochondrial sulfide oxidizing pathway is sulfide:quinone oxidoreductase (SQR, Figure 7). SQR is a member of the protein family of disulfide oxidoreductases, which has been identified in all life domains [73]. It is a membrane-associated protein located at the periplasmic side of the cytoplasmic membrane in prokaryotes and at the inner mitochondrial membrane in eukaryotes. Although no structure of a eukaryotic SQR has thus far been obtained, crystallographic structures of prokaryotic SQRs have been reported [74–76], enabling the construction of a structural model of human SQR (Figures 8(a) and 8(b)). Several structural and amino acid sequence features linked to sulfide oxidation and quinone reduction have allowed for the classification of the SQR family into six subclasses, with human SQR classified as a type II SQR [77]. The main features of type II SQRs, particularly as compared to type I SQRs, are essentially the lack of any extended loops, the poor conservation of quinone-interacting residues, and the substitution of the conserved cysteine covalently linking the FAD cofactor for a tyrosine (Tyr_170_ according to human SQR numbering), which probably establishes a covalent link with FAD in human SQR via an 8-*α*-*O*-tyrosyl bond [77]. SQRs can be monomeric or assemble into dimers or trimers of ~50 kDa subunits [78], each harboring a cysteine disulfide in its active site and a noncovalently bound flavin adenine dinucleotide (FAD) moiety implicated in electron transfer [77]. In mammals, SQR couples H_2_S oxidation to the reduction of coenzyme Q, with the concomitant transfer of a sulfur atom to an acceptor that has been proposed to be either sulfite- (SO_3_^2−-^) yielding thiosulfate (S_2_O_3_^2−^) [79–81] or reduced glutathione (GSH) yielding glutathione persulfide (GSSH) [82]. Although the protein displays a higher catalytic efficiency using as cosubstrate sulfite (*k*_cat_/*K*_*M*_ = 2.5 × 10^6^ M^−1^ · s^−1^) as compared to GSH (*k*_cat_/*K*_*M*_ = 1.6 × 10^4^ M^−1^ · s^−1^) [83], at physiological concentrations of both cosubstrates, GSH was suggested to be the preferential sulfur acceptor [82–84], at variance from [79–81]. In contrast to the mammalian protein, bacterial SQRs directly release the oxidized sulfur as a soluble polysulfide with up to ten sulfur atoms, instead of reacting with a sulfur acceptor [75, 85]. The reaction mechanism of SQR has been investigated with the isolated protein not only in detergent solution [80, 82, 84] but also after incorporation into nanodiscs [83], where slightly higher catalytic rates have been measured. The postulated mechanism involves (i) reaction of the active site cysteine disulfide with H_2_S associated with persulfidation of Cys_379_ (human SQR numbering) to form CysSSH and release of the Cys_201_ thiolate that in turn forms a charge transfer complex with FAD, (ii) transfer of the sulfane sulfur from the persulfidated Cys_379_ to the acceptor with the concomitant reduction of FAD to FADH_2_, and (iii) oxidation of FADH_2_ by coenzyme Q. According to kinetic data, the sulfane sulfur transfer step is the rate-limiting one in the reaction.

**Figure 9:**
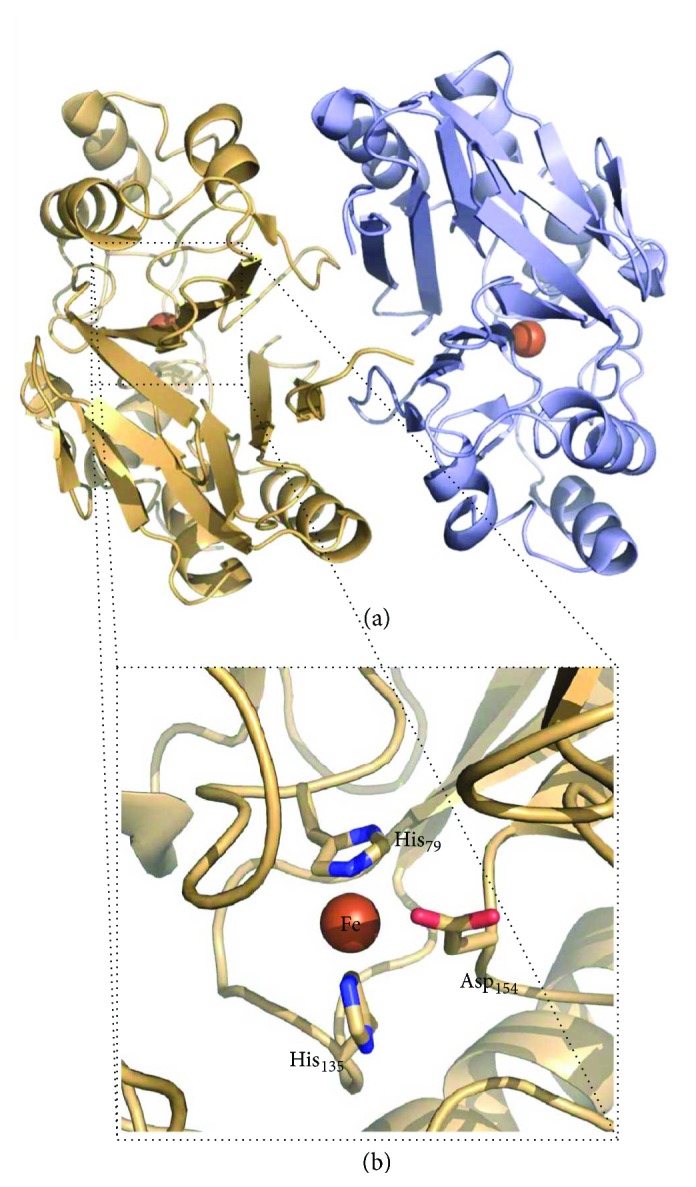
Crystallographic structure of human persulfide dioxygenase (ETHE1). (a) Cartoon representation of human ETHE1 homodimer (PDB ID: 4CHL; 2.61 Å resolution). (b) Zoom-in into the mononuclear nonheme iron catalytic site. Iron ligands (His_79_, His_135_, and Asp_154_; human ETHE1 numbering) are represented in sticks. Figure generated with PyMol 1.8.2.0 [238].

The next step in the sulfide oxidation pathway is catalyzed by ETHE1 (Figure 7). Mutations in the gene encoding this protein were found to result into an autosomal recessive disorder, known as ethylmalonic encephalopathy, characterized by a severe clinical picture and leading to death during early childhood [86, 87]. The crystallographic structure of human ETHE1 was recently solved (Figure 9) [88]. ETHE1 is a dimeric enzyme, each monomer comprising an *αββα* fold structurally related to dizinc classical metallo-*β*-lactamases, harboring a mononuclear nonheme iron octahedrally coordinated by two histidines, one aspartate, and three water molecules [88]. The enzyme is proposed to catalyze the oxygenation of the sulfane sulfur atom in the GSSH released from SQR, yielding sulfite [55]. It is important to note that one O_2_ molecule is consumed in the reaction.

GSSH and sulfite are further converted into thiosulfate and GSH by rhodanese (Rhod, Figure 7). No structural studies of the human enzyme have been reported, yet a structural model can be obtained based on the ~90% identical bovine homologue (Figures 8(c) and 8(d)) [89]. This protein has a redox-active cysteine (Cys_248_) in the active site and is highly promiscuous in that it can effectively react with several different substrates. Yet, at physiologically relevant substrate concentrations, the most likely reaction catalyzed by rhodanese was suggested to be the transfer of a sulfur atom from GSSH to sulfite to form thiosulfate [82]. Sulfite oxidase (SOx, Figure 7) catalyzes the last step in the sulfide oxidation pathway. SOx is a multidomain cytochrome *b*_5_ with a molybdenum cofactor [90] and a heme mediating the intramolecular electron transfer from sulfite to cytochrome *c*. Concomitantly, an oxygen atom is supplied by an H_2_O molecule and sulfate (SO_4_^2−^) forms as the end-product of the reaction [90].

Overall, the mitochondrial sulfide-oxidizing pathway couples the oxidation of H_2_S to thiosulfate and sulfate with the injection of electrons into coenzyme Q, leading to consumption of 0.79 O_2_ per H_2_S molecule oxidized, as reported by Goubern and coworkers [61], which is compatible with the predicted value of 0.75. Indeed, based on the postulated architecture of the mitochondrial sulfide oxidation pathway presented above, the oxidation of 2 H_2_S molecules (involving 2 electrons) is expected to require the consumption of 1.5 O_2_ molecules (0.5 by CcOX plus 1 by ETHE1). As O_2_ is required for sulfide removal, the efficacy of mitochondrial sulfide oxidation is expected to depend on O_2_ availability, clearly vanishing under anoxic conditions. Evidence for a decline of mitochondrial sulfide oxidation at lower O_2_ concentrations was preliminarily provided in [69] and, then, substantiated in a later study [63]. To be noted that at very low O_2_ concentrations (down to 0.73 ± 0.04 *μ*M), given a fixed amount of sulfide, a faster onset of cell respiration inhibition was observed at decreasing O_2_ concentrations [69], which is consistent with a lower efficacy of sulfide removal but also with a higher control of the respiratory electron transfer chain by CcOX at low O_2_ concentration.

Our knowledge of H_2_S catabolism and, particularly, of its implication in human physiology and pathophysiology is still rather elusive. Current views of the mitochondrial sulfide oxidation pathway are yet rather patchy and represent a matter of debate. It is likely that the physiological function of this pathway goes well beyond the mere removal/detoxification of H_2_S. The impact of this pathway on cell bioenergetics under specific physiological or pathological conditions needs to be better established, as well as its ability to produce bioactive sulfur metabolites (e.g., thiosulfate) whose physiological role has only recently begun to emerge. Finally, it can be anticipated that the mitochondrial sulfide oxidation pathway plays a key role in mediating the intricate interplay between sulfide, O_2_, and the other gasotransmitters, but more studies are needed in this direction.

## 3. Signaling Mediated by Hydrogen Sulfide

The relevance of hydrogen sulfide in mammalian physiology results essentially from its role as a second messenger that transduces signals by interaction with target proteins. The initial classical view of H_2_S-mediated signaling initially merely considered the inhibitory properties of hydrogen sulfide towards respiratory CcOX (detailed below) leading to a state of suspended animation. Presently, the signal transducing function of hydrogen sulfide is known to occur via at least two major mechanisms, namely, through (i) interaction with protein metal centers, particularly heme moieties, and (ii) protein persulfidation (detailed below).

### 3.1. H_2_S and Heme Proteins

**Figure 10:**
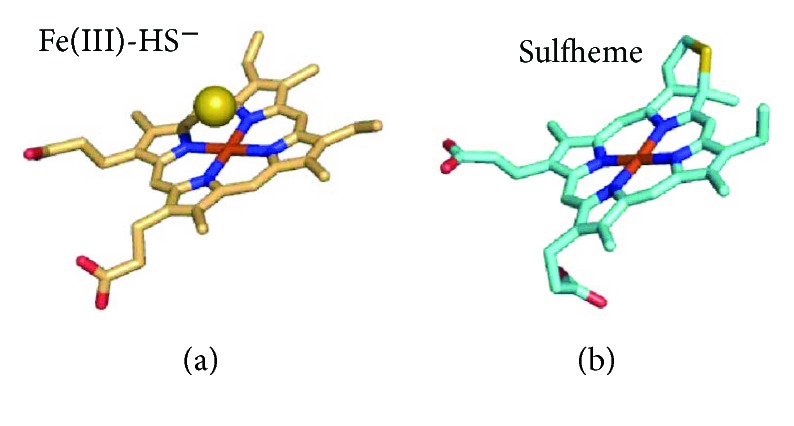
Structure of sulfide-reacted heme moieties in globins. (a) Structure of ferric heme moiety (light orange sticks) in human hemoglobin with bound HS^−^ (yellow sphere); PDB entry: 5UCU [115]. (b) Structure of sulfheme moiety (light blue sticks) generated by reaction of horse myoglobin and sulfide, resulting in sulfmyoglobin; PDB entry 1YMC [239].

Besides being able to promote thiol persulfidation, H_2_S can react with protein hemes. As reviewed in Bianco et al. [91], the mechanism and fate of the reaction of H_2_S with a heme protein largely depend on several factors, such as the redox and ligation state of the heme iron, the environment in the heme pocket, the protonation state of bound sulfide, and the presence/absence of O_2_ or reducing agents in solution. Heme-Fe(III) can bind H_2_S as such or as HS^−^ (Figure 10). Stability of one or the other heme adduct generated by reaction with hydrogen sulfide (heme-Fe(III)-H_2_S or a heme-Fe(III)-HS^−^) depends on the protein residues in the heme surroundings and, particularly, on the presence of either basic residues able to accept protons from bound H_2_S to yield the heme-Fe(III)-HS^−^ species or, alternatively, nonpolar residues stabilizing the fully protonated state of the ligand. Owing to its reducing character, bound sulfide can reduce the heme iron, yielding a heme-Fe(II)-HS· radical adduct that can further react with excess HS^−^/HS· or O_2_/H_2_O leading, respectively, to formation of polysulfides or thiosulfate. In contrast to these reactions, H_2_S can also promote a covalent modification of the heme, yielding the so-called sulfheme derivative with a sulfur atom incorporated into one of the porphyrin pyrrole rings (Figure 10). The mechanism of sulfheme formation is still unclear. Sulfheme can be formed by reaction of H_2_S with either the ferrous oxygenated (heme-Fe(II)-O_2_ [92]) or the ferryl heme (heme-FeIV=O [93, 94]). However, also in the former case the reaction requires H_2_O_2_ and thus, likely, the formation of higher valent heme iron intermediates [95, 96]. As reviewed in [5, 91, 95–97], the reactions above have been described by several groups working on numerous hemeproteins, including, among others: globins from mammals, invertebrates, and bacteria; heme-based sensor proteins of diatomic molecules such as O_2_, NO, and CO; cytochrome *c* oxidase; catalase; and peroxidases (lactoperoxidase, myeloperoxidase and thyroid peroxidase).

Because heme proteins can usually react not only with H_2_S but also with NO, CO, and O_2_, they can play a key role in mediating the crosstalk between these gaseous ligands, namely, controlling each other's biological function. A paradigm of this concept is CcOX. The enzyme, highly reactive with O_2_, is indeed a recognized target for all three gasotransmitters (NO, CO, and H_2_S). Each of these species can inhibit CcOX, though with markedly different kinetics and mechanisms [29]. While CO can only bind to the fully reduced heme *a*_3_-Cu_B_ active site, leading to competitive inhibition of the enzyme [98], CcOX inhibition by NO can proceed through two alternative reaction pathways (reviewed in [99–104]): an O_2_-competitive pathway, favoured at lower O_2_ tension and higher electron flux, leading to a nitrosyl adduct of CcOX with NO bound to ferrous heme *a*_3_, and an O_2_-uncompetitive one, prevailing at lower electron flux and higher O_2_ tension, whereby an inhibited adduct of enzyme forms with nitrite bound at ferric heme *a*_3_ [29, 105–107]. The mechanism of CcOX inhibition by H_2_S has not been investigated as thoroughly as for NO and CO. Differently from these two species, H_2_S does not bind ferrous heme *a*_3_. Based on the postulated mechanism, when the enzyme in turnover with O_2_ is exposed to H_2_S, oxidized or reduced Cu_B_ is the primary target site in CcOX. Afterwards, sulfide is thought to be transferred intramolecularly to the nearby ferric heme *a*_3_, resulting in enzyme inhibition [30]. H_2_S-inhibited CcOX thus exhibits one sulfide molecule bound to ferric heme *a*_3_ [108, 109] and, possibly, a second one bound to cuprous Cu_B_, as revealed by electron paramagnetic resonance (EPR) spectroscopy [110]. Regardless of the exact molecular mechanism, CcOX inhibition by H_2_S was demonstrated to be relatively fast (initial rate constant of 2.2 × 10^4^ M^−1^·s^−1^, as measured at pH 7.4 with the enzyme in turnover with O_2_ and cytochrome *c* [30]), effective (*K*_*i*_ = 0.2 *μ*M at pH 7.4), independent of O_2_ concentration, and reversible [52]. Similarly to other heme proteins, CcOX inhibition by H_2_S was found to be greatly dependent on pH. Acidic conditions remarkably enhance the inhibitory action of sulfide, and consistently, as the pH shifts from 8.05 to 6.28, *K*_*i*_ decreases from 2.6 *μ*M to 0.07 *μ*M [111]. The enhanced efficacy of sulfide inhibition at lower pH suggests that sulfide binds to CcOX either as H_2_S (e.g., in fully protonated state) or as HS^−^ with the concomitant protonation by a protein residue. This is consistent with the active site of the enzyme being located in a nonpolar environment, therefore facilitating the binding of electroneutral or proton-neutralized anionic species [112].

H_2_S can also act as a reducing substrate for CcOX [30, 111, 113], when the enzyme is in the so-called pulsed state [30], but the reaction products are yet to be characterized. By reacting with H_2_S at relatively low concentrations, heme *a*_3_ in the active site of the enzyme is promptly reduced and, by further reacting with O_2_, leads to a ferryl species with optical features indistinguishable from those of the “**P**” catalytic intermediate [30]. Afterwards, the “**F**” ferryl intermediate spontaneously forms on a longer timescale, but it is unclear if the reaction results from autoreduction or electron donation from residual sulfide [30]. CcOX is therefore in principle able to catalyze the oxidative breakdown of H_2_S, but the reaction is probably too slow to be physiologically relevant, particularly when compared with the high H_2_S-metabolizing activity of SQR.

Another group of proteins whose reactivity with H_2_S has been investigated in detail is the globin family. These studies focused primarily, but not exclusively, on human hemoglobin (Hb) and myoglobin (Mb). The reaction of sulfide with Hb has long been known to lead to irreversible covalent conversion of the protein heme to sulfheme (reviewed in [96]); as described above, the modification consists in the incorporation of a sulfur atom into the heme pyrrole B. The resulting protein derivative, named “sulfhemoglobin,” exhibits a characteristic green color and lower O_2_ affinity as compared to the native protein. Accumulation of sulfhemoglobin is therefore associated to toxicity, leading to a rare pathological condition known as “sulfhemoglobinemia.” A similar reactivity has been demonstrated also for Mb, leading to formation of “sulfmyoglobin” (Figure 10). Whereas sulfhemoglobinemia likely represents a form of sulfide toxicity, methemoglobinemia, that is, the accumulation of ferric hemoglobin (metHb) in the blood, has been suggested to protect against sulfide toxicity in mice [114]. In these *in vivo* studies, 2–4 mol of H_2_S was inferred to be inactivated per mol of metHb [114]. This superstoichiometric value suggests that metHb is able not only to bind H_2_S but also to promote its catalytic breakdown. Consistently, under aerobic conditions metHb was recently demonstrated to bind H_2_S, oxidizing it to a mixture of thiosulfate and polysulfides [21]. The reaction, though proceeding at relatively low rates (3 min^−1^ in the presence of 0.5 mg·mL^−1^ metHb, at pH 7.4 and 25°C [21]), could be of physiological relevance, as Hb-rich red blood cells lack mitochondria and therefore cannot dispose H_2_S via the mitochondrial sulfide-oxidizing pathway. Sulfide is a relatively low-affinity ligand for metHb: at pH 7.4 and 37°C, it binds with a *k*_on_ of 3.2 × 10^3^ M^−1^·s^−1^ and dissociates from the oxidized heme with a *k*_off_ of 0.053 s^−1^, yielding a *K*_*D*_ of 17 *μ*M (whose value may be overestimated, given that the H_2_S species represents only a fraction of sulfide at pH 7.4). The rate constant for H_2_S binding to metHb was found to increase with decreasing pH, and on this basis, H_2_S rather than the HS^−^ species, more abundant at pH 7.4, initially binds to the heme. After binding to the heme iron, H_2_S is proposed to deprotonate to HS^−^. The postulated Fe(III)-HS^−^ species has been recently detected by X-ray crystallographic analysis of the sulfide-reacted metHb (Figure 10) [115].

Possible mechanisms for polysulfide and thiosulfate formation by metHb have been proposed in [21]. Interestingly, the newly formed polysulfide remains bound to the protein upon heme iron reduction by methionine sulfoxide reductase (MSR). In contrast, it reacts with physiologically relevant, low-molecular-weight (LMW) thiol compounds, such as reduced glutathione (GSH) or cysteine (CysSH), yielding the corresponding persulfides, GSSH and CysSSH, that are eventually released into the solution. In light of the high intracellular concentration of GSH, Hb can act as a source of GSSH that in turn could serve as a persulfide donor in protein persulfidation (detailed below). Therefore, based on its intricate chemistry with sulfide, depending on conditions, Hb can mediate sulfide toxicity via formation of sulfhemoglobin or be protective by promoting sulfide disposal, a particularly relevant function in red blood cells lacking mitochondria. Furthermore, Hb can be viewed as a source of physiologically relevant sulfide oxidation products, namely, thiosulfate and glutathione persulfide (GSSH), whose impact on human physiology is emerging. A somewhat different scenario occurs in atherosclerotic lesions, particularly when they are infiltrated by red blood cells. Indeed, in those lesions, oxidized forms of Hb with the heme in the ferric and ferryl state accumulate and mediate toxicity by promoting radical reactions leading to formation of cross-linked Hb species and lipid modification. Under these circumstances, H_2_S was recently reported to exert protective effects, acting as a reductant of ferryl Hb [116].

Another heme protein whose reactivity with sulfide was investigated in depth is human leukocyte myeloperoxidase (MPO). Based on recent studies [117, 118], upon reaction with sulfide, the enzyme does not lead to formation of the sulfheme derivative, at variance with other heme proteins. Of interest, MPO was reported to catalyze the oxidation of sulfide to sulfane sulfur species both in the presence and in the absence of H_2_O_2_ [117, 118]. The reaction involves as a central intermediate compound III, a resonance form between the ferrous/dioxygen (Fe(II)-O_2_) and ferric/superoxide (Fe(III)-O_2_·^−^) complex, it is facilitated by ascorbate or SOD and might be of physiological relevance as the formed sulfane sulfur species were proven to oxidize protein cysteine residues to their corresponding per/polysulfide derivatives [117].

### 3.2. Persulfidation and Persulfide (Bio)chemistry

**Figure 11:**
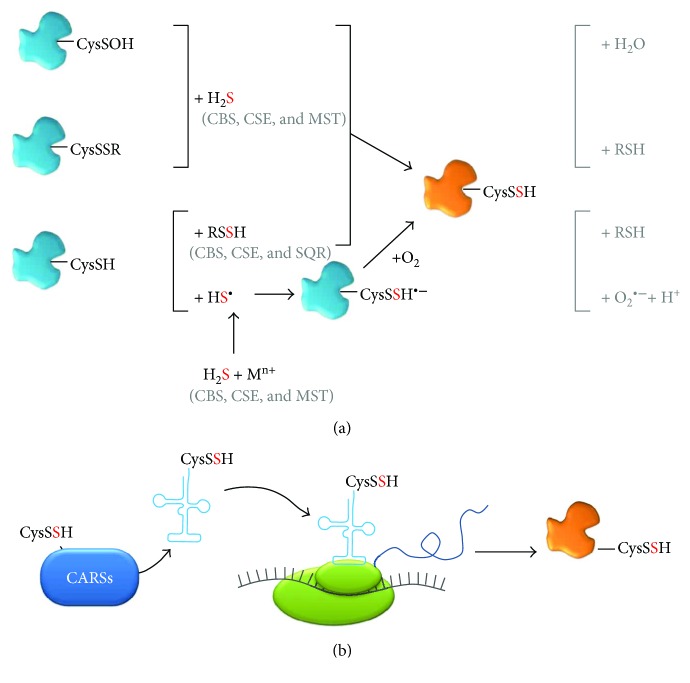
Reactions leading to protein persulfidation. Scheme depicting the most plausible *in vivo* reactions leading to cysteine persulfidation in target proteins, through (a) posttranslational modification of cysteine residues or (b) cotranslational incorporation of CysSSH via CysSSH-bound tRNA derived from cysteinyl-tRNA synthetases (CARSs). (a) Proteins with oxidized cysteine residues (CysSSR and CysSOH) can react directly with H_2_S (derived from its synthesizing enzymes CBS, CSE, and MST) to yield a protein cysteine persulfide (CysSSH). Proteins with reduced cysteine residues (CysSH) can react with free LMW persulfides (RSSH, such as glutathione persulfide and cysteine persulfide, products of H_2_S biosynthetic—CBS, CSE, and MST—or catabolic—SQR—enzymes) or with the HS· radical formed by reaction of H_2_S with metal centers (M^n+^). The resulting protein-bound cysteine perthiyl radical (CysSSH·^−^) can then react with oxygen to yield CysSSH and superoxide anion. (b) Generation of protein cysteine persulfides through cotranslational incorporation of CysSSH. Mammalian cysteinyl-tRNA synthetases (CARSs, dark blue box, which also synthesize free CysSSH from cysteine) catalyze the synthesis of tRNA-bound cysteine persulfides (light blue line), which incorporate CysSSH into the nascent polypeptide (dark blue line) at cysteine sites upon translation (ribosome depicted in green and mRNA in grey).

As mentioned above, many of the signaling functions attributed to H_2_S have been shown to be tightly associated with formation of persulfides through the modification of specific cysteine side chains from target proteins, often involving free reactive LMW persulfides (RSSH), particularly cysteine persulfide (CysSSH) and glutathione persulfide (GSSH). Polysulfides are also considered as potentially relevant from a physiological viewpoint, as reviewed elsewhere [119]. However, because they are contaminants of most inorganic H_2_S donors employed in research and therefore possibly responsible for some of the effects attributed to H_2_S, their (bio)chemistry is still far from being fully understood and will not be detailed herein. The relevance of persulfidation is attested by the fact that deficiency in persulfidated proteins has been associated with different pathologies, including cardiovascular and neurodegenerative (Parkinson's) diseases [120, 121]. Besides its signaling function, persulfidation may also constitute a mechanism to prevent thiol oxidation or electrophilic modification and irreversible damage [122]. The ability of LMW persulfides to readily donate one electron and form a stable perthiyl radical (RSS·), unreactive towards oxygen or NO, led to the proposal of a possible role in redox processes [123]. Recently, the oxidation of cysteine persulfides to perthiosulfenic acid (CysSSOH) observed in different proteins as a consequence of NADPH oxidase activation led to the proposal of this modification as an additional redox-based signaling event [124]. The chemical and biochemical properties, biosynthesis and natural occurrence, analytical detection methods, and cellular longevity of persulfides and polysulfides have all been covered in excellent articles and reviews (e.g., [3, 6, 120, 121, 125–129]). As in other fields focused on reactive molecules, there seem to be controversies as to which species and reactions are more plausible and relevant from a chemical-to-physiological viewpoint. Presently, there is however a consensus that H_2_S cannot react directly with protein cysteine thiols. Protein persulfidation, in order to occur, has special requirements both at a “local” (the environment surrounding the target cysteine) and a “global” (redox status, free glutathione/cysteine/H_2_S availability) level. Currently, although several possibilities for the formation of persulfidated proteins through posttranslational modification can be envisaged, only four appear to be more plausible (Figure 11(a)). Two of these possibilities involve previous oxidation of the protein target cysteine thiol to a sulfenic moiety (CysSOH), for example, by reaction with hydrogen peroxide, or to a disulfide (CysSSR) by reaction with an oxidized sulfur-containing LMW molecule, for example, oxidized glutathione (GSSG). Both of these oxidized cysteine residues can then be targeted directly by H_2_S, resulting in a persulfidated protein. Reduced cysteine residues (e.g., protein thiols) can be modified to their persulfide derivatives through reaction with a free LMW persulfide (RSSH), such as GSSH or (homo)cysteine persulfide (derived from the H_2_S biosynthetic or catabolic pathway [125, 126]), or via reaction with HS·, generated upon reaction of H_2_S with metal centers, particularly oxidized protein heme iron. The protein cysteine thiol reaction with HS· should yield a transient CysSSH·^−^ that, upon reaction with oxygen, yields the protein-bound CysSSH and superoxide anion, similar to that reported for HSSH·^−^ [3, 130]. In addition to posttranslational persulfidation, it has been quite recently posited that in mammals a major source of persulfidated (and polysulfidated) proteins *in vivo* are actually cysteinyl-tRNA synthetases (CARSs, particularly the mitochondrial CARS2), through cotranslational incorporation of cysteine persulfide or polysulfide at cysteine sites into the nascent polypeptide [131] (Figure 11(b)). From a cellular signaling perspective, the advantage of protein persulfidation by H_2_S and/or persulfides is that the resulting species can be reverted to the thiol moiety in different ways, such as direct reaction with reduced glutathione (GSH) or through the thioredoxin system [126], thus affording a reliable switching modification.

As for the LMW persulfides, which are key molecules in protein persulfidation, they are increasingly coming into the limelight due to their intrinsic reactivity (e.g., [125]). Until recently, the two major sources of these persulfides, particularly cysteine persulfide (CysSSH) and glutathione persulfide (GSSH), were considered the enzymes involved in H_2_S metabolism. CysSSH is readily generated by the H_2_S-synthesizing CBS and CSE, using cystine (CysSSCys) as substrate [125]. Notably, in the course of this reaction, longer polythiolated products with catenated sulfur atoms, such as CysSSSH (synthesized by both CBS and CSE) and CysSSSSH (synthesized by CSE), have been detected. The enzymatic generation of these species further results in appreciable amounts of oxidized polysulfide species like CysSSSCys, CysSSSSCys, and CysSSSSSCys. The newly formed CysSSH and longer cysteine persulfides can then react with reduced glutathione to yield GSSH and GS(S)_n_H. It should however be noted that, at physiologically relevant substrate concentrations, H_2_S rather than CysSSH is expected to be the major product of the transsulfuration pathway enzymes [132], also taking into account that in the cytoplasm, cysteine and reduced glutathione are much more abundant than their oxidation products cystine and GSSG [132]. Recently, MST was also recognized as a source of CysSSH and GSSH, as well as of longer polysulfides, related with its ability to generate other RSS, such as hydrogen persulfide (H_2_S_2_) and hydrogen trisulfide (H_2_S_3_) [133, 134]. MST-derived persulfides and polysulfides have been associated with higher cellular levels of protein-bound sulfane sulfur [134, 135]. The MST-catalyzed CysSS_(n)_H and GSS_(n)_H production was demonstrated with recombinant MST (wild-type and site-directed variants), MST-expressing COS cells lysates, and mouse brains (cell suspensions and whole brains) [133], showing that in the brain LMW persulfides and protein persulfidation are essentially generated via MST and not CBS or CSE [133]. A principal role in CysSSH (and CysSS_(n)_H) synthesis has been recently proposed for mammalian CARSs, particularly the mitochondrial isoform CARS2 [131]. The same report suggests that mitochondria are the key cellular compartments where CysSSH is formed before being released into the cytosol to exert its effects. It is also posited that whereas CARSs may be the major source of CysSSH under physiological conditions, CBS and CSE may still act as major players in CysSSH synthesis in pathophysiological conditions associated with oxidative and electrophilic stress with concomitantly increased cystine concentrations [125, 131, 132].

Besides the LMW persulfides derived from the H_2_S-synthesizing enzymes CBS, CSE, and MST [125, 132, 133], GSSH is a metabolic intermediate of the mitochondrial sulfide-oxidizing pathway [126] (Figure 7). Indeed, GSH has been suggested as the preferential sulfur acceptor in the H_2_S oxidation reaction catalyzed by SQR, yielding GSSH [82]. This persulfide is, in turn, the preferential substrate (with sulfite as cosubstrate) for rhodanese to generate thiosulfate as the final oxidation product of sulfide oxidation, together with sulfate [82].

Regardless of how LMW persulfides are generated, their reactivity prompts them to be major signal-transducing species linked to H_2_S (patho)physiology. In a nutshell, persulfides are stronger nucleophiles than their thiol counterparts are (yet displaying also a weak electrophilic character). Despite this assumption, only recently it was clearly demonstrated by Cuevasanta et al. [136] that a human serum albumin persulfide derivative displays 20-fold increased reactivity with respect to its thiol counterpart. Persulfides have lower p*K*_a_ values than the analogous thiols, thus existing mostly in their anionic nucleophilic RSS^−^ form [3, 6]. Conversely, their weak electrophilicity is exhibited solely in their protonated RSSH form. Interestingly, both the outer sulfhydryl and inner sulfenyl sulfurs have electrophilic character, the reaction occurring on either one depending on factors such as steric hindrance or acidity of the leaving group [3]. The ambiguous nucleophilic/electrophilic nature of LMW persulfides, while at the basis of their physiological relevance, accounts for their instability. These species indeed quickly decay or react with other species, particularly oxidants, hampering their characterization both *in vitro* and *in vivo*. This notwithstanding, the molecular details and functional consequences of persulfidation at specific cysteine residues have been demonstrated for a number of targets and shown to regulate several physiological processes (reviewed in, e.g., [127]), such as glycolysis [26], ER stress [137], apoptosis [138], vasorelaxation, and vasodilation [139] (detailed in Table 1). Regarding the latter, H_2_S was the third gasotransmitter, following NO and CO, to be regarded as an endothelium-derived relaxing factor (EDRF) and as an endothelium-derived hyperpolarizing factor (EDHF), thus regulating vasodilation (detailed below). Despite the relatively few examples available in the literature, protein persulfidation is likely prevalent, yet technically challenging to be detected due to the intrinsic reactivity of persulfides. Using an adaptation of the biotin switch assay modified to detect persulfidated rather than nitrosated proteins, Mustafa et al. reported that 25–50% of hepatic proteins are persulfidated [26]. The extent of protein persulfidation *in vivo* has been further demonstrated by increasingly elaborate and quantitative methodologies [125, 140–143]. It is expected that the number of identified new targets of protein persulfidation will continuously increase, pairing this posttranslational modification with other highly relevant cellular switches.

## 4. Interplay between Gasotransmitters

### 4.1. Brief Historical Account of Gasotransmitters

**Figure 12:**
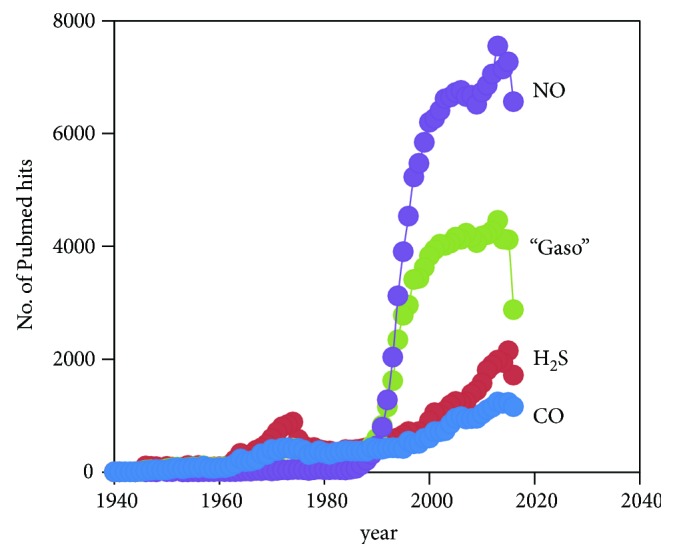
Literature on gasotransmitters, H_2_S, NO, and CO. Number of articles retrieved from Pubmed queries with the following terms: “hydrogen sulfide” (H_2_S), “gasotransmitters” (“gaso”), “nitric oxide” (NO), and “carbon monoxide” (CO).

The term “gasotransmitters” [144] has entered the biochemistry and physiology jargon to designate essentially the three small molecules gaseous in nature with demonstrated roles in signal transduction: nitric oxide (NO; although the formally correct abbreviation should be NO·, herein NO will be used for simplicity), carbon monoxide (CO), and hydrogen sulfide (H_2_S). Arguably, molecular oxygen (O_2_), although not synthesized endogenously in mammalian cells, could be considered a gasotransmitter due to its gaseous nature, reactivity, and physiological relevance. Besides, O_2_ is a substrate of the mammalian enzymes that synthesize NO and CO, so O_2_ levels dictate the bioavailability of the latter. Gasotransmitter-mediated signaling is an intricate and integrated process involving not only NO, CO, H_2_S, and O_2_ but also the collective pool of reactive species (reactive oxygen species (ROS), reactive nitrogen species (RNS), and reactive sulfur species (RSS) resulting from their cross-reactions, establishing a so-called reactive species interactome (RSI) [145]. Irrespectively of the identity of the gasotransmitter molecule or derived species, its signaling mode of action can be narrowed down essentially to two possibilities: interaction with metal centers or modification of cysteine residues in proteins, both resulting either in the activation or in the inactivation of the target protein. The three gasotransmitters NO, CO, and H_2_S share a common history in that they were initially merely regarded as toxic and poisonous molecules, until the discovery that virtually all life forms, from bacteria to man, are endowed with dedicated synthesizing and detoxifying enzymes and produce or scavenge NO, CO, and H_2_S to accomplish specific functions. Indeed, in the late 1980s and early 1990s, major advances concerning the role of NO in the regulation of cardiovascular function led to NO being considered Molecule of the Year in 1992 and to the Nobel Prize in Physiology or Medicine being awarded in 1998 to Robert Furchgott, Ferid Murad, and Louis Ignarro Jr “for their discoveries concerning nitric oxide as a signaling molecule in the cardiovascular system” [146]. One of the key findings was the identification of the endothelium-derived relaxing factor (EDRF) as NO [147, 148]. This observation, which revealed beneficial rather than deleterious effects of an otherwise considered toxic molecule, triggered a subfield of biology dedicated to signaling by gaseous second messengers. Indeed, besides other regulatory roles attributed to NO, similar findings were demonstrated for CO, which was also shown to have a role in vasorelaxation [149]. The same function came to be assigned to H_2_S [17], which also led it to be labeled as an EDRF [127]. The multiple functions of gasotransmitters in human physiology are well attested by the “explosion” of interest that arose since the discovery of NO as a signaling molecule in the mid-1980s, CO in the mid-1990s, and H_2_S in the 2000s. This is well illustrated by carrying out a Pubmed search querying “gasotransmitters,” “nitric oxide,” “carbon monoxide,” and “hydrogen sulfide” (Figure 12). Altogether, the three gasotransmitters regulate, often through similar molecular processes, vasodilation, energy metabolism, redox homeostasis, apoptosis, cell cycle, reproduction, neuronal function, and so on, and dysregulation of the metabolism and/or levels of NO, CO, or H_2_S is associated with several pathological conditions, from cardiovascular and neurodegenerative diseases to diabetes and cancer, among others.

A surprising element in gasotransmitter history was the finding that mammals, among many other organisms, encode specialized enzymes that synthesize these previously considered noxious gaseous molecules. NO is enzymatically produced by NO synthases, of which three isoforms have been described and historically classified according to their location or regulation, yet presently considered to be ubiquitous and constitutively expressed: inducible NO synthase (iNOS), endothelial NO synthase (eNOS), and neuronal NO synthase (nNOS) (reviewed, e.g., in [150]). Regardless of the isoform and its localization or regulation, NO synthases catalyze the conversion of arginine and O_2_ into NO and citrulline, using electron equivalents derived from NADPH. Besides being synthesized on demand by NO synthases, NO is endogenously generated (i) under hypoxic conditions through nitrite reduction by several heme proteins (including hemoglobin, as reviewed in [151]), (ii) in the digestive tract as an intermediate of bacterial nitrogen metabolism (particularly in the mouth and the gut), and (iii) chemically from nitrite under the acidic environment in the stomach (reviewed, e.g., in [152]). As for CO, it is generated by either isoform of heme oxygenase, HO-1 and HO-2, which catalyze the conversion of heme, with O_2_ as cosubstrate, into biliverdin and CO, employing NADPH-derived electron equivalents (reviewed, e.g., in [153]). H_2_S synthesis is catalyzed by CBS, CSE and MST, as described above.

As mentioned before, it has become increasingly clear that no gasotransmitter should be regarded as an isolated entity with an independent cellular function and lifespan. Rather, gasotransmitters interact with each other not only by direct cross-reactions that generate other reactive species but also by having cooperative or opposite molecular and cellular consequences towards their targets. Moreover, each gasotransmitter in many ways is able to modulate the synthesis of the others, which represents an additional intricate level of regulation. An example of this modulatory action is described below, concerning the regulation of CBS-catalyzed H_2_S production by NO and CO.

### 4.2. Chemical Bases of the Interplay between H_2_S and NO

The biological effects of H_2_S and NO are intimately interconnected. The interplay between these two gaseous molecules takes place through numerous mechanisms acting at different levels (reviewed in [154]). Many studies aimed at gaining insight into the chemical foundation of the interaction between H_2_S and NO. An extensive analysis has been carried out of the reactivity of sulfide with several NO metabolites, including nitrite [155], peroxynitrite [156, 157], and nitrosothiols [158–163], and with NO releasers such as nitroprusside [163–167]. Despite these efforts, the picture is currently far from being completely clear. As discussed in [5, 168–171], some remarkable discrepancies have indeed emerged, mainly regarding the nature of the species generated in these reactions and their suitability as signaling molecules under physiological conditions.

The first S/N hybrid species suggested to form by reaction of nitrosothiols (e.g., *s*-nitrosoglutathione (GSNO)) with sulfide was thionitrous acid/thionitrite (HSNO/ONS^−^) [161]. In line with previous studies by Whiteman et al. [172, 173], Filipovic et al. [161] reported the formation of HSNO by reaction of H_2_S with either GSNO or acidified nitrite, as well as by investigation of the reaction of NO with HS· generated by pulse radiolysis. In addition to HSNO, the reaction of GSNO with sulfide was found to yield also a yellow product assumed to be a mixture of polysulfides. Of interest regarding its potential physiological implications, HSNO was proposed to serve not only as a source of both NO and nitroxyl anion (NO^−^) but also as a donor of nitrosonium ion (NO^+^) to thiols, thereby promoting transnitrosation reactions even across biological membranes [161]. On these bases, HSNO was suggested to be a key species in the interplay between H_2_S and NO, sitting at the crossroad between the H_2_S and NO signaling pathways.

This view was challenged in subsequent studies by Cortese-Krott et al. [145, 155, 159, 160, 169, 174], who failed to detect HSNO accumulation during the reaction of GSNO with H_2_S and claimed HSNO to be too short-lived to accumulate and be identified under physiological conditions, in agreement with Munro and Williams [175] and Williams [176]. Compared to other physiologically relevant nitrosthiols, HSNO was indeed suggested to be much more unstable as it undergoes a facile isomerization by hydrogen shift, leading to formation of four distinct isomers. The yellow product generated by reaction of GSNO with H_2_S initially described in [161] was assigned by Cortese-Krott et al. [159] to nitropersulfide (SSNO^−^), a compound discovered and chemically characterized in the '80s [177]. The same research groups reported later [160] that, under physiologically relevant conditions, the reaction between sulfide and NO generates not only SSNO^−^ but also polysulfides and SULFI/NO [ON(NO)SO_3_^−^], an additional bioactive compound consisting of an adduct of two molecules of NO with sulfite. Based on these and further investigations [174], Cortese-Krott et al. reported that SSNO^−^ is stable in the presence of thiols and cyanides and is able to release NO upon decomposition, targeting the Keap1/Nrf2 redox system, among other possible ones. Overall, this led to the proposal that SSNO^−^, rather than HSNO, plays a key role in the interplay between NO and H_2_S under physiological conditions [159]. In line with this proposal, SSNO^−^ was reported to be very stable under anaerobic conditions, to decay slowly (half life of about 40 minutes) in aqueous buffer under aerobic conditions, and to directly react with deoxygenated metHb with a rate constant of 11 M^−1^·s^−1^, yielding ferrous nitrosyl Hb (HbNO) presumably via NO release [178]. In contrast, by characterizing pure crystalline SSNO^−^ prepared as a bis(triphenylphosphine)iminium (PNP^+^) salt according to [179], Filipovic et al. reported that SSNO^−^ is a highly unstable species, leading to rapid decomposition with formation of HNO/NO^−^ in the presence of water, light, or acid and producing HSNO by reaction with H_2_S, cyanide, and GSH [180, 181].

From the observations reported above, it is clear that additional investigations are needed to reconcile the discrepancies present in the literature and gain further insight into the chemical foundation of the interplay between NO and H_2_S.

### 4.3. Modulation of H_2_S Metabolism by NO and CO

Besides the chemical reactions that define new players in gasotransmitter-mediated signaling, there is an intricate web of cross-regulatory mechanisms whereby each gasotransmitter regulates the homeostasis of the others. While part of that cross-regulation may occur at the transcriptional and translational level, herein we will mainly cover the molecular mechanisms that result from direct interactions between the gasotransmitters and their target proteins, focusing on the modulation by NO and CO of H_2_S biosynthesis and breakdown.

#### 4.3.1. Modulation of H_2_S-Generating Enzymes by NO, CO, and Derived Species

The most thoroughly studied H_2_S-generating enzyme in terms of regulatory mechanisms has certainly been CBS. In what regards its modulation by the other gasotransmitters NO and CO, CBS regulation is centered at its noncatalytic heme bound to the N-terminal domain. This interplay between gasotransmitters results essentially from the fact that H_2_S production by CBS is reversibly inhibited by NO and CO. The direct effect of this inhibitory mechanism would be that local increases in NO or CO result in decreased H_2_S synthesis, which has been demonstrated in different cellular models and tissues [182–185]. However, recent evidence suggests that CBS inhibition may in fact result in overall increased H_2_S production, due to a metabolic switch in the transsulfuration pathway (Figure 1(a)) [11, 186]. In this perspective, inhibiting CBS deviates the transsulfuration branch from cysteine to H_2_S production via CSE, which has a higher H_2_S-synthesizing activity, particularly using the accumulated homocysteine also resulting from CBS inhibition [11, 186]. Moreover, the pathophysiological significance of CBS inhibition by gasotransmitters may extend beyond “merely” affecting H_2_S homeostasis. CBS acts as a metabolic hinge determining the ratio between the remethylation and transsulfuration steps of the methionine cycle (Figure 1(a)). CO inhibition of CBS in various cancer cells has been shown to affect the methylation state of 6-phosphofructo-2-kinase/fructose-2,6-biphosphatase 3 (PFKFB3), suppressing 6-phosphofructo-2-kinase, resulting in a shift of glucose from glycolysis to the pentose phosphate pathway [34, 187].

The regulatory heme-binding domain may be considered a hallmark of CBS evolution, since it is absent from CBS from prokaryotes or unicellular eukaryotes. The low-spin hexacoordinate *b*-type heme is axially bound to the His_65_ imidazole and Cys_52_ thiolate moieties (human CBS numbering; Figure 4(b)). Besides its regulatory role, the CBS heme has been proposed to have a structural stabilizing effect and contribute to improve CBS folding [188]. After initial submission of this review, a quite interesting observation has been reported, which will once again stir the field of CBS heme regulation. Kumar et al. observed that the N-terminal residues 1–40, so far absent from all CBS crystallographic structures, constitute an intrinsically disordered region that is able to bind heme with Cys and His as axial ligands, in a cysteine-proline-based motif [189]. This observation, deserving further exploration, constitutes an additional level of complexity regarding all the literature on the CBS heme regulation (see below).

**Figure 13:**
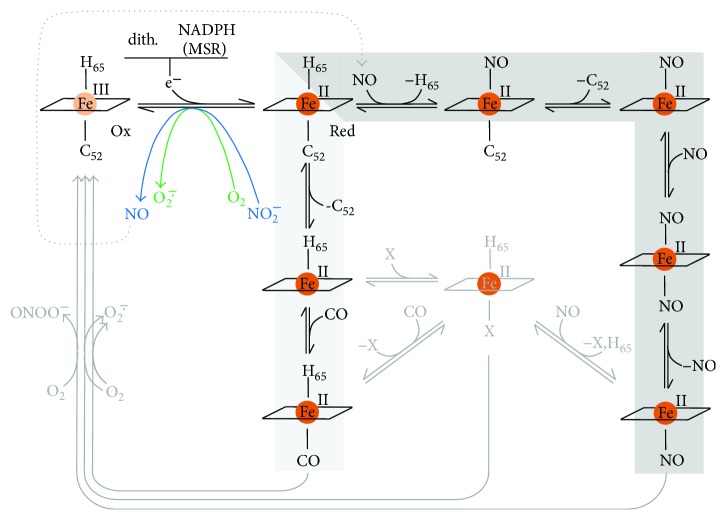
Mechanisms of heme reduction and ligand binding to human CBS.

The heme regulatory role is essentially related to its redox state and the nature of its endogenous and exogenous ligands. The initial designation of the CBS heme as a redox sensor stemmed from its sensitivity to reducing conditions. Indeed, heme reduction by excess sodium dithionite at 37°C slowly (>20 minutes) inactivates the enzyme, which has been assigned to a ligand switch process in which one of the endogenous ligands of heme iron is replaced by another as yet unknown ligand (Figure 13) [190]. The resulting species has been termed “C424” due to its characteristic UV-visible absorption spectrum, in which the Soret band obtained for reduced CBS with its *λ*_max_ at 449 nm is shifted to a lower-intensity band centered at 424 nm. Irrespectively of the bound endogenous ligands, once reduced, the heme is able to bind the gasotransmitters CO and NO, which results in reversible enzyme inhibition (Figure 13) [191–195]. Although the low reduction potential (−350 mV, [196]) measured for the CBS heme raised some doubts about the occurrence of reduced CBS heme *in vivo*, the human enzyme methionine synthase reductase (MSR) has been shown to be able to catalyze CBS reduction *in vitro* at the expense of NADPH oxidation. Moreover, MSR is able to generate both the ferrous-CO and the ferrous-NO species of human CBS [191, 197]. Despite the significant distance between the heme and PLP cofactors (~11 Å from edge to edge and ~20 Å from the Fe ion to the pyridoxine ring), communication between them is ensured by a network of molecular interactions occurring at helix 8 (depicted by the yellow arrow in Figure 4(b)) [198, 199]. While at the PLP end of helix 8 the residues Thr_257_ and Thr_260_ establish hydrogen bonds with the PLP phosphate group, at the heme extremity of this helix Arg_266_ is in electrostatic contact with the heme ligand Cys_52_ thiolate moiety. The relevance of these residues in the allosteric communication between the active (PLP) and regulatory (heme) sites has been further demonstrated in clinically relevant variants [199, 200].

The kinetic characterization of the interplay between the three gasotransmitters centered at CBS has been made possible by taking advantage of the distinct characteristic UV-visible absorption spectra displayed by the heme cofactor in different oxidation and ligation states. Most studies on the CBS redox state and ligand reactivity have thus employed static and time-resolved (stopped-flow) absorption spectroscopy [192–195, 199–204].

The reaction of reduced CBS with CO has been thoroughly characterized *in vitro* and shown to result in a hexacoordinate species in which the endogenous Cys52 ligand has been replaced by CO, with no evidence for stable intermediates. Indeed, the reaction is limited by the slow dissociation of Cys_52_ (0.003–0.017 s^−1^ [192, 195, 197, 204]), as confirmed by laser flash photolysis and time-resolved Raman spectroscopy [192]. Equilibrium titrations revealed that CO binds to ferrous CBS according to two dissociation constants (*K*_*d*1_ = 0.7–1.5 *μ*M and *K*_*d*2_ = 45–68 *μ*M; *C*_50_ ≈ 30 *μ*M), which has been assigned to anticooperativity between paired monomers [192] or differences in the heme microenvironment [194]. The inhibitory effect of CO binding to the ferrous heme in human CBS is characterized by *K*_*i*_ values of 5.6–9.5 *μ*M [194, 204].

Recently, our groups have reported that a clinically relevant variant with a mutation in the N-terminal domain, p.P49L, displays a markedly higher affinity for CO with respect to WT, while showing an essentially WT-like behavior in other functional and structural terms [203]. While this mutation may induce increased flexibility of the loop containing both heme endogenous ligands, the overall higher proneness to become inhibited by CO may represent a new pathogenic mechanism in classical homocystinuria. Current efforts to develop alternative therapies for classical homocystinuria have essentially focused on chemical and pharmacological chaperones to recover misfolded protein variants originating from missense mutations [205–211] and recently on the development of a PEGylated CBS for enzyme replacement therapy [212–214].

NO binding to reduced CBS is remarkably faster and displays much higher affinity than CO binding. However, initial reports on the reaction between ferrous CBS and NO indicated otherwise. Very low affinity for NO was reported (*K*_*d*_ = 281 *μ*M), employing a high excess of sodium dithionite to keep CBS reduced throughout the NO titrations and using a slow NO releaser (diethylamine NONOate) as NO source [193]. Under these conditions, free NO, either unreacted or dissociated from the ferrous-NO CBS adduct, reacts with the excess dithionite and leads to an overestimation of the dissociation constants, as later shown even with minute amounts of excess dithionite [195]. Recently, our groups have shown that NO binding to ferrous CBS occurs with an affinity compatible with a physiological role of NO in controlling CBS function [195]. An apparent *K*_*d*_ of 0.23 *μ*M was determined at the lowest dithionite concentration used in that study, which implies that the actual affinity is even higher than measured. Contemporarily, a *K*_*d*_ = 30 *μ*M was determined and still was considered an upper limit for this dissociation constant, as it was measured using NADPH and human MSR as reduction system, therefore involving multiple equilibria (NADPH ↔ MSR, MSR ↔ CBS, and CBS ↔ NO) [191]. As compared to CO, NO binding to reduced CBS not only occurs with markedly higher affinity but is also orders of magnitude faster, as investigated by stopped-flow absorption spectroscopy. Differently from CO, NO binding results in a pentacoordinate ferrous-NO adduct, where both endogenous ligands have been displaced and a single NO molecule is bound. No reaction intermediates have been detected upon mixing NO with reduced CBS, at least within the time resolution of the stopped-flow equipment. Moreover, also differently from CO, NO binding proceeds with rate constants linearly dependent on NO concentration (up to 800 *μ*M), that is, unlimited by dissociation of the endogenous ligands, yielding a bimolecular rate constant of ~8 × 10^3^ M^−1^·s^−1^ (at 25°C). In line with the higher affinity and faster binding of NO with respect to CO, NO dissociation from ferrous CBS is slower than CO dissociation. In mechanistic terms, since NO binding is not rate-limited by the displacement of Cys_52_ (which instead limits CO binding), it has been proposed that NO may initially displace the His_65_ heme ligand (Figure 13). In turn, the absence of evidence for a hexacoordinate intermediate suggests that Cys_52_ is immediately displaced upon NO binding. Finally, although it has been established that the final NO adduct is a pentacoordinate species, it remains to be demonstrated whether NO ends up on the His_65_ or Cys_52_ side of the heme, since both residues are supposed to dissociate from the heme iron. It should be noted that if NO ends up on the Cys52 side, the binding mechanism must involve the formation of a transient bis-NO intermediate (Figure 13). Irrespectively of the mechanistic details, the kinetic and spectroscopic investigations of CBS reduction and CO and NO binding have determined parameters compatible with an *in vivo* role of this regulatory mechanism.

The reaction of reduced CBS with O_2_ was also investigated by stopped-flow absorption spectroscopy and shown to be reasonably fast, occurring with a bimolecular rate constant of ~1 × 10^5^ M^−1^·s^−1^ (25°C) [202], that is, approximately 10-fold faster than the reaction with NO. Although this reaction reverts CBS back to its active ferric state, it also generates superoxide anion as a reaction product, which may have damaging effects. This oxidation process has indeed been reported to involve a partial heme loss. Superoxide anion promptly reacts with NO (at rates close to the diffusion limit) to yield the powerful oxidant peroxynitrite. Interestingly, it has been shown that CBS heme not only is inactivated by peroxynitrite but also may be a source of it. The reaction of oxidized CBS with peroxynitrite has been shown to result in enzyme inactivation with an IC_50_ of 150 *μ*M, occurring according to a bimolecular rate constant of 2.4–5 × 10^4^ M^−1^·s^−1^ and leading to nitration of Trp_208_, Trp_43_, and Tyr_223_, as well as damaging the heme moiety [215]. Intriguingly, a recent report demonstrated that the reaction between ferrous-NO CBS and O_2_, previously shown to revert the heme to its ferric state, yields peroxynitrite [201] (Figure 13). Moreover, the reaction of reduced CBS with nitrite may itself constitute a source of NO [191, 201], which further adds to the complex heme-mediated regulation of H_2_S synthesis by CBS (Figure 13).

#### 4.3.2. Effect of AdoMet on CO/NO Binding and Inhibition of H_2_S Production by CBS

CBS continues to surprise researchers in the field since regulation of the protein activity not only is intricate but also at times appears odd. It has long been established that AdoMet is an activator of CBS, increasing its enzymatic activity by 2- to 5-fold. Moreover, AdoMet binding also has a stabilizing effect, particularly under “normal” (nonpathological) conditions [216]. Recently, the structural details of this regulatory mechanism have been elucidated. While the C-terminal regulatory domain from one monomer blocks the substrate entry site in the catalytic core of an adjacent monomer, AdoMet binding to the C-terminal domain (also called Bateman module) causes a domain shift and leads to the association of two C-terminal domains in a disk-like form, unblocking the substrate entrance and thus “activating” (or derepressing) the enzyme [44, 46]. The relevance of this regulatory mechanism is well attested by the fact that some homocystinuric patients express CBS variants with mutations in the C-terminal domain, that are naturally activated (more active than WT) and have limited or absent AdoMet response as the molecular basis of disease [217, 218]. While working on the kinetic characterization of NO and CO binding to human CBS, we noticed that AdoMet has the intriguing effect of eliciting faster and tighter binding of both CO and NO, thus making the enzyme more prone to inhibition by either gasotransmitter [204]. This observation is directly related to the binding of exogenous ligands, since AdoMet had been shown to have no effect on the heme redox properties [196]. Under the same reaction conditions, AdoMet makes CO inhibition >10 times more potent than in its absence (*K*_*i*(CO, +AdoMet)_ = 0.7 *μ*M versus *K*_*i*(CO, −AdoMet)_ = 9.5 *μ*M) [204]. The kinetic details of this functional effect have been investigated, and it was shown that AdoMet increases CO affinity by ~5-fold and association kinetics by ~10-fold, whereas the effect on NO binding is less drastic (~2-fold higher affinity with respect to AdoMet-free CBS). The latter observation also enforces the previous mechanistic proposal that CO and NO “attack” different sides of the heme. Recently, this observation has been extended to a clinically relevant variant, p.P49L, which displays the same AdoMet response in terms of becoming more sensitive to CO, while being slightly impaired in the AdoMet activation of H_2_S production, compared to WT [203]. Irrespectively of the mechanistic details, the AdoMet-induced enhanced CO and NO binding appears as an intricate regulatory mechanism which adds to the complexity of CBS regulation.

#### 4.3.3. Modulation of Sulfide Oxidation by NO

Whereas the effect of CO and NO on H_2_S biosynthesis has been investigated in detail, much scarcer is the information on the effect of these two gaseous molecules on H_2_S catabolism. Because CO and NO, similarly to cyanide, can both potently inhibit CcOX through different mechanisms (see above), relatively low concentrations of these gasotransmitters are expected to affect mitochondrial sulfide oxidation. Following cell exposure to CO or NO, a transient inhibition of CcOX is indeed expected to occur and lead to electron accumulation in the mitochondrial respiratory chain, eventually impairing quinol oxidation. As a result, SQR-mediated oxidation of sulfide is expected to be impaired as long as CcOX inhibition persists. Accordingly, addition of the NO releaser MAMA NONOate (≥20 *μ*M) or cyanide (260 *μ*M) to colonocytes was recently reported to inhibit cellular respiration and its stimulation by 20 *μ*M NaHS [219]. Furthermore, it is worth noting that long-term intraperitoneal or intravenous administration of nitroglycerine in rats was shown to induce decreased rhodanese activity and diminished levels of sulfane sulfur in liver extracts [220]. The observed inhibition of rhodanese was proposed to arise from *s*-nitrosation of the catalytic cysteine in the protein active site. A cysteine in the isolated rhodanese from bovine liver was indeed shown to be susceptible to *s*-nitrosation by the NO donors *s*-nitroso-*N*-acetylpenicillamine (SNAP) and GSNO, leading to enzyme inhibition [220].

The preliminary information reported above unveils an additional level at which the interplay between the gasotransmitters can be established. A full description of these molecular mechanisms awaits further investigations.

#### 4.3.4. Modulation of NO and CO Synthesis by Hydrogen Sulfide

In light of the crosstalk between gasotransmitters, hydrogen sulfide has been shown to have a role in the control of NO and CO bioavailability under specific (patho)physiological conditions.

**Figure 14:**
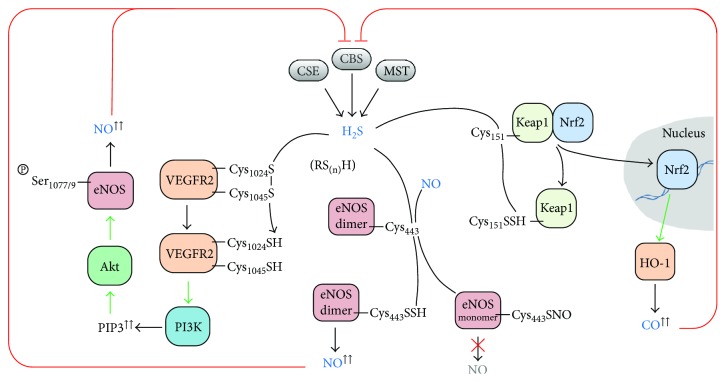
H_2_S-mediated modulation of nitric oxide and carbon monoxide biosynthesis. Hydrogen sulfide produced by CBS, CSE, and MST, and the related per- and polysulfide species (RS_(n)_H), can modulate NO and CO bioavailability through different mechanisms. Left, representation of sulfide-mediated enhanced eNOS activity: H_2_S reduces VEGFR2 Cys_1024_-Cys_1045_ disulfide, resulting in activation of PI3K; the accumulated PIP3 increases Akt-mediated phosphorylation of eNOS at Ser_1077_ and/or Ser_1079_, enhancing its NO-synthesizing activity. Center, direct persulfidation of eNOS Cys_443_ sustains the protein catalytic activity by competing with Cys_443_*s*-nitrosation, which causes dissociation of the active dimeric into the inactive monomeric form. Right, modulation of CO biosynthesis through increased expression of HO-1, mediated by the Keap1/Nrf2 pathway. Persulfidation of Keap1 Cys_151_, either by reaction of H_2_S with oxidized Cys_151_ or of reduced Cys_151_ with H_2_S-derived HS·, causes its dissociation from Nrf2, which can then translocate to the nucleus, where it enhances heme oxygenase HO-1 expression, thus resulting in higher CO levels that may become inhibitory for CBS.

As mentioned above, CO is generated by either isoform of heme oxygenase, HO-1 and HO-2 [153]. Several lines of evidence point to a role of hydrogen sulfide in increasing the expression of heme oxygenase, particularly HO-1, and consequently CO levels (mostly evaluated from the abundance of carboxylated hemoglobin), thereby exerting either protective or deleterious effects (e.g., [221–227]). The underlying regulatory mechanism is proposed to involve the Keap1/Nrf2 system. In particular, persulfidation of Keap1 Cys_151_, by reaction either of H_2_S with oxidized Cys_151_ or of reduced Cys_151_ with H_2_S-derived HS· (Figure 11(a)), causes the protein dissociation from Nrf2 that, in turn, translocates into the nucleus, ultimately resulting in increased HO-1 transcription and protein levels (Figure 14) [225]. Another possibility whereby CO production may be modulated resides in the fact that a thiol/disulfide redox switch comprising three Cys-Pro signatures in HO-2 regulates heme binding [228, 229]. The sensitivity of this thiol/disulfide switch to the cellular redox state and possibly to the intracellular sulfide levels may afford another level of H_2_S-mediated control of CO levels. Interestingly, the beneficial health effects of certain garlic-derived organosulfur compounds, particularly diallyl disulfide and trisulfide, have been proposed to be related with the modulation of CO production by H_2_S via the Keap1/Nrf2 pathway [230–232].

The control of NO biosynthesis by hydrogen sulfide has been investigated in more detail and appears to occur at the transcriptional, translational, and posttranslational levels, involving other signaling pathways, besides a direct inhibition of all NOS isoforms by H_2_S. The abundance of data in the literature highlights the fact that the effect of H_2_S on the expression and activity of each NOS isoform can vary—sometimes yielding conflicting results—with the model organ, tissue, or cell type investigated and with the nature, concentration, and potency of the employed sulfide donor. Moreover, whereas in some studies “only” the levels of NO (or nitrite/nitrate) are measured as a function of sulfide exposure, other studies further investigate the NO-mediated cellular and physiological effects of sulfide exposure.

Regulation of eNOS by H_2_S has been thoroughly investigated. In different cell types and under different conditions and stimuli, eNOS activation in response to sulfide exposure seems to be mediated by the PIP3/Akt (phosphatidylinositol 3,4,5-trisphosphate/serine/threonine-specific protein kinase) pathway (Figure 14). The molecular mechanism is proposed to involve the reduction by H_2_S of the vascular endothelial growth factor receptor 2 (VEGFR2) Cys_1024_-Cys_1045_ disulfide that in the oxidized state represses its receptor activity. This H_2_S-induced disulfide reduction thus prolongs the VEGFR2 activity and consequently the phosphatidylinositol-4,5-bisphosphate 3-kinase (PI3K) activity, yielding an increase in PIP3 levels (which may also be influenced by persulfidation of Cys_124_ in the phosphatase and tensin homolog (PTEN)). The resulting increased Akt activation leads to enhanced eNOS phosphorylation at Ser_1177_ and/or Ser_1179_, thereby increasing the NO-synthesizing activity. Another level of sulfide-mediated eNOS regulation concerns the direct persulfidation at Cys_443_ (Figure 14). Whereas dimerization appears to be a prerequisite for optimal eNOS activity, *s*-nitrosation of Cys_443_ induces dimer dissociation into the virtually inactive monomeric form. Sulfide-mediated persulfidation of this residue actively competes with *s*-nitrosation, thereby maintaining eNOS in the active homodimeric form. Direct *in vitro* inhibition of recombinant eNOS by H_2_S is characterized by an extremely high (nonphysiological) IC_50_ of 170 *μ*M [233]. Regarding the sulfide-mediated modulation of eNOS expression, various reports have either shown a down- or upregulation of eNOS mRNA and protein levels in response to H_2_S exposure. These somehow contradictory results obtained for different model systems (animals, organs, and cells) rule out the establishment of a clear trend, induction versus repression, for sulfide modulation of eNOS expression.

Besides its direct *in vitro* inhibition at nonphysiological H_2_S concentrations (IC_50_ = 210 *μ*M) [233], sulfide-mediated modulation of iNOS is reported to occur mainly through regulation of its expression. Similarly to eNOS, initial contradictory results indicated either an increase or a decrease in iNOS-mediated NO production in response to sulfide exposure [234]. This controversy was addressed by comparatively investigating the effects of different H_2_S donors [235], showing that the rate of H_2_S release by the donors is a key determinant for the downstream effects. Subsequent investigations on various models commonly assigned an inhibitory effect of sulfide on iNOS mRNA expression and consequently iNOS-mediated NO production, thereby contributing to the establishment of H_2_S as an anti-inflammatory molecule (reviewed, e.g., in [154]).

Currently, the information on nNOS modulation by H_2_S is scarce. Besides its inhibition at high nonphysiological sulfide concentrations (IC_50_ = 130 *μ*M) [233, 236], nNOS expression seems to be either insensitive to sulfide exposure or decreased upon overexpression of the H_2_S-synthesizing CBS in hypertensive (but not normotensive) rats [237].

Altogether, the multiple molecular mechanisms whereby each of the three gasotransmitters may control the bioavailability of the others highlight the intricate signaling mechanisms that govern mammalian (patho)physiology and offer valuable possibilities for pharmacological targeting of each pathway.

## 5. Concluding Remarks

Hydrogen sulfide evolved from being merely perceived as a toxic and poisonous molecule to a prominent signaling molecule in mammalian physiology, joining nitric oxide and carbon monoxide as the triad of gasotransmitters. The last couple of decades witnessed an accumulation of data showing that (i) H_2_S is endogenously synthesized and catabolized by specialized enzymes and kept under a tight control through multiple intricate regulatory mechanisms, (ii) the signaling functions attributed to H_2_S are mainly linked to the interaction with and modification of target proteins, via reaction with metal centers (mostly in heme proteins) and through cysteine persulfidation that alters protein structure and function, (iii) dysregulation of H_2_S homeostasis is at the basis of several pathologies, including cardiovascular and neurodegenerative diseases and cancer, and (iv) possible mechanisms underlying a functional crosstalk between the three gasotransmitters have been unveiled. The clear link between H_2_S metabolism and homeostasis with human health has triggered a strong interest in the discovery and development of pharmacological (synthetic and naturally derived) compounds, either to release H_2_S or to modulate the H_2_S metabolism enzymes. Despite the accumulated knowledge on hydrogen sulfide biochemistry over the years, all a contribution as this one can amount to be, particularly concerning a field in its infancy, is just a still picture of a fast moving *body*. Indeed, during the writing of this manuscript, several pieces of information even challenging previous observations arose in the literature, further contributing to the complexity of this quickly progressing field.

## Figures and Tables

**Table 1:** Protein targets for cysteine persulfidation and molecular and cellular consequences.

Protein	Target cysteine	Physiological process	Consequences of persulfidation	Refs.
Protein Tyr phosphatase 1B (PTP1B)	Cys_215_	ER stress response	PTP1B is inhibited, enhancing PERK^(a)^-mediated phosphorylation of eIF2*α*^(b)^, suppressing protein translation.	[137]
p65 subunit of nuclear factor *к*B (NF-*к*B)	Cys_38_	NF-*κ*B signaling; apoptosis	Stimulates binding of p65 to ribosomal protein S3, resulting in increased translation of prosurvival genes.	[138]
Inwardly rectifying potassium channel subunit Kir6.1	Cys_43_	Vasorelaxation and vasodilation	Enhances channel activity through decreased affinity for ATP and increased binding of PtdIns(4,5)P_2_^(c)^.	[139]

^a^Protein kinase RNA-like ER kinase; ^b^eukaryotic translational initiation factor 2*α*; ^c^phosphatidylinositol-4,5-bisphosphate.

## Abbreviations

3-MP: 3-Mercaptopyruvate
AdoHcy: *S*-Adenosyl-L-homocysteine
AdoMet: *S*-Adenosyl-L-methionine
ADT-OH: 5-(4-Hydroxyphenyl)-3*H*-1,2-dithiole-3-thione
Akt: Serine/threonine-specific protein kinase
AOAA: Aminooxyacetic acid
AP39: [10-Oxo-10-(4-(3-thioxo-3*H*-1,2-dithiol-5yl)phenoxy)decyl) triphenylphosphonium bromide]
ATP: Adenosine triphosphate
CARSs: Cysteinyl-tRNA synthetases
CAT: Cysteine (or aspartate) aminotransferase
CBS: Cystathionine *β*-synthase
CcOX: Mitochondrial cytochrome *c* oxidase (complex IV)
CO: Carbon monoxide
CSE: Cystathionine *γ*-lyase
CysSOH: Cysteine residue with modified thiol to sulfenic acid
CysSSH: Cysteine persulfide
CysSS_(n)_H: Cysteine polysulfide
CysSSOH: Cysteine perthiosulfenic
CysSSR: Cysteine residues with modified thiol to disulfide
DHLA: Dihydrolipoic acid
EDHF: Endothelium-derived hyperpolarizing factor
EDRF: Endothelium-derived relaxing factor
eIF2*α*: Eukaryotic translational initiation factor 2*α*
eNOS: Endothelial nitric oxide synthase
EPR: Electron paramagnetic resonance spectroscopy
ETHE1: Persulfide dioxygenase, or ethylmalonic encephalopathy protein 1
FAD: Flavin adenine dinucleotide
GAPDH: Glyceraldehyde 3-phosphate dehydrogenase
GSNO: *S*-Nitrosoglutathione
GSSH: Glutathione persulfide
GSS_(n)_H: Glutathione polysulfide
Hb: Hemoglobin
H_2_S: Hydrogen sulfide
H_2_S_2_: Hydrogen persulfide
H_2_S_3_: Hydrogen trisulfide
HO-1: Heme oxygenase 1
HO-2: Heme oxygenase 2
HS^−^: Hydrosulfide
HSNO/ONS^−^: Thionitrous acid/thionitrite
iNOS: Inducible nitric oxide synthase
Keap1: Kelch-like ECH-associated protein 1
Kir6.1: Inwardly rectifying potassium channel subunit Kir6.1
LMW: Low-molecular-weight
metHb: Ferric hemoglobin
Mb: Myoglobin
MSR: Methionine synthase reductase
MST: 3-Mercaptopyruvate sulfurtransferase
NADPH: Nicotinamide adenine dinucleotide phosphate (reduced form)
NF-*κ*B: Nuclear factor *κ*B
nNOS: Neuronal nitric oxide synthase
NO: Nitric oxide (NO·)
NO^−^: Nitroxyl anion
NO^+^: Nitrosonium ion
Nrf2: Nuclear factor erythroid 2-related factor 2
PERK: Protein kinase RNA-like ER kinase
PI3K: Phosphatidylinositol-4,5-bisphosphate 3-kinase
PIP3: Phosphatidylinositol 3,4,5-trisphosphate
PLP: Pyridoxal 5′-phosphate
PNP^+^: Bis(triphenylphosphine)iminium
PPG: Propargylglycine
PtdIns(4,5)P_2_: Phosphatidylinositol-4,5-bisphosphate
PTEN: Phosphatase and tensin homolog
PTP1B: Protein Tyr phosphatase 1B
Rhod: Rhodanese
RNS: Reactive nitrogen species
ROS: Reactive oxygen species
RSS: Reactive sulfur species
RSS·: Perthiyl radical
S^2−^: Sulfide
SAHH: *S*-Adenosyl-L-homocysteine hydrolase
SMCs: Vascular smooth muscle cells
SOx: Sulfite oxidase
SQR: Sulfide:quinone oxidoreductase
SSNO^−^: Nitropersulfide
Tom20: Translocase of the outer membrane 20
TPP^+^: Triphenylphosphonium
Trx: Thioredoxin
VEGFR2: Vascular endothelial growth factor receptor 2.
